# Extracellular Vesicles in Neonatology: Have We Found the Magic Bullet?

**DOI:** 10.3390/biology15191734

**Published:** 2026-10-01

**Authors:** Paraskevi Papadogeorgou, Andreas G. Tsantes, Serena Valsami, Sotirios P. Fortis, Anastasios G. Kriebardis, Vasiliki Mougiou, Zoi Iliodromiti, Theodora Boutsikou, Nicoletta Iacovidou, Rozeta Sokou

**Affiliations:** 1 Neonatal Department, Aretaieio Hospital, National and Kapodistrian University of Athens, 11528 Athens, Greece; vassouli@hotmail.com (V.M.); ziliodromiti@med.uoa.gr (Z.I.); theobtsk@gmail.com (T.B.); niciac58@gmail.com (N.I.); sokourozeta@gmail.com (R.S.); 2 Laboratory of Haematology and Blood Bank Unit, “Attiko” Hospital, School of Medicine, National and Kapodistrian University of Athens, 12462 Athens, Greece; andreas.tsantes@yahoo.com; 3 Microbiology Department, “Saint Savvas” Oncology Hospital, 11522 Athens, Greece; 4 Laboratory of Hematology and Blood Bank Unit, Aretaieio Hospital, Medical School, National and Kapodistrian University of Athens, 11528 Athens, Greece; svalsami@med.uoa.gr; 5 Laboratory of Reliability and Quality Control in Laboratory Hematology (HemQcR), Department of Bio-Medical Sciences, School of Health & Caring Sciences, University of West Attica (UniWA), 12243 Athens, Greece; sfortis@uniwa.gr (S.P.F.); akrieb@uniwa.gr (A.G.K.)

**Keywords:** extracellular vesicles, exosomes, microparticles, neonates

## Abstract

Extracellular vesicles (EVs) represent nanoparticles released by numerous cell types and exerting distinct biological roles. Extracellular vesicles are enclosed in a lipid layer and contain proteins, lipids, nucleic acids, carbohydrates or even mitochondria. The fundamental utility of EVs is intercellular communication, while their implication in various biological processes, such as inflammation and hemostasis, is well-documented. Growing evidence indicates the significant usefulness of EVs as biomarkers in certain prematurity-related diseases, like bronchopulmonary dysplasia (BPD) and necrotizing enterocolitis (NEC). Moreover, many in vitro and in vivo studies have been undertaken in order to explore the potential therapeutic impact of EVs in BPD, NEC and retinopathy of prematurity (ROP), some of them yielding promising results. The present narrative review aims towards the summarization of the existing data on the application of EVs in diverse neonatal disorders, such as BPD, NEC, ROP, sepsis, multi-organ failure, coagulopathy and brain injury. The widespread investigation of EVs in neonatology and their potential implementation in clinical practice may lead towards a revolutionary approach and management of neonatal diseases, as well as an innovative perception of prematurity.

## Simple Summary

Extracellular vesicles (EVs) represent small cargo-bearing vesicles released by cells, which have been of increasing concern in recent years due to their unique properties and characteristics. Although EVs were previously considered systems for discarding cellular waste and intercellular communication, nowadays, it is well-recognized that EVs are able to shuttle functional cargo from cells and can be used as diagnostic biomarkers, as well as therapeutic tools. The EVs utility has been trialed in many fields of medicine, like immunomodulation, cancer, regenerative therapy, pharmacology and hopefully neonatology. The endogenous characteristics of EVs, such as their ability to cross biological barriers and their involvement in paracrine signaling of stem cells, render them appealing therapeutic modalities that may be applied as standalone therapies. The EVs functionality can be further enhanced by bioengineering methods to harbor specific pharmaceutical substances or even genes, augment their stability and modify their surface epitope. Although clinical translation of EVs’ utility in neonatology is still in its infancy and many aspects require assessment and verification, EVs application holds great promise and may represent a breakthrough advancement of medicine. The current review aims to integrate and thoroughly analyze the available studies exploring the possible usefulness of EVs as diagnostic biomarkers and therapeutic modalities in neonatology, as well as the future perspectives on the field.

## 1. Introduction

The term “extracellular vesicles—EVs” is used in order to describe the membrane-enclosed vesicles secreted naturally by both prokaryotic and eukaryotic cells, delimited by a lipid bilayer and containing proteins, lipids, nucleic acids, carbohydrates or even mitochondria [1,2,3,4].

The first mention of EVs was made as early as the late 19th century by Horder, who identified them by microscopic methods and described them as “dancing bodies” or “blood dust” [5]. Later, in 1967, Wolf P. isolated a pellet of EVs from fresh plasma freed of intact platelets after ultracentrifugation [6]. Extracellular Vesicles (EVs) do not have the ability to replicate because they do not have a functional nucleus, but they are distributed in all human bodily fluids, such as blood, urine and saliva [1,7]. A variety of terms, including exosomes, microvesicles, microparticles, ectosomes, oncosomes, and apoptotic bodies, have historically been used to describe extracellular vesicular populations based on their presumed biogenesis, size, or cellular origin. However, according to the MISEV2023 guidelines, the generic term “extracellular vesicle” (EV) is preferred unless the specific biogenesis or subcellular origin of a vesicle population has been reliably demonstrated. Extracellular vesicles (EVs) are defined as particles released from cells that are delimited by a lipid bilayer and cannot replicate independently [8]. Importantly, exomeres and supermeres are considered non-vesicular extracellular particles (NVEPs) rather than EV subtypes because they lack a lipid bilayer [9].

Historically, cell-derived vesicles have also been named according to their cell or tissue of origin, including dexosomes, prostasomes, matrix vesicles, and synaptic vesicles [10,11,12,13]. Current MISEV2023 recommendations favor operational EV terminology based on measurable characteristics, including size, density, biochemical composition, or cellular origin. Accordingly, EV populations may be described operationally as small EVs (sEVs), commonly <200 nm, or large EVs (lEVs), commonly >200 nm, while recognizing that these size thresholds are not absolute and depend on the characterization method. Biogenesis-based terms such as “exosome” or “ectosome” should be used only when the corresponding subcellular origin has been adequately demonstrated. In particular, “sEV” and “exosome” should not be used interchangeably, as small EV populations may comprise vesicles originating through different biogenesis pathways [8].

Originally, EVs have been considered a cellular mechanism to jettison wasted materials, but growing evidence clarifies that EVs represent a major component of intercellular communication both in health and disease [4,14,15,16,17]. The full spectrum of EV action is wide and incorporates several biological processes, such as inflammation, hemostasis and immunity [18,19,20]. Moreover, EVs are able to be transferred both to close and distal cells in the extracellular space, serving as a mechanism of paracrine and autocrine regulation. A novel utilization of exosomes is also the delivery of therapeutic agents to injured cells [21]. Furthermore, transmission of nucleic acids, such as RNA, between cells has revised the mechanisms of gene regulation, since EVs provide post-transcriptional changes and post-translational regulation in gene expression in target cells [17,22,23]. Particularly, microRNA (miRNA) represents a non-coding RNA of 19–24 nucleotides in size that leads to degradation of messenger RNAs and post-transcriptional silencing of gene expression in the target cell [24,25]. Moreover, the beneficial role of EVs in tissue healing and neuronal rejuvenation has shifted the research attention towards innovative approaches of tissue engineering [26]. Finally, EVs are implicated in the pathophysiology and progression of several disorders, such as cancer, diabetes and chronic renal failure [20].

Extracellular vesicles (EVs) represent a fundamental element of the cell secretome and represent fingerprints of their cell of origin [24]. The EV cargo includes several bioactive molecules, like proteins, lipids, cellular metabolites, messenger RNA (mRNA), miRNA and DNA. Hormones, cytokines and coagulation factors are also transferred by microvesicles. The protein cargo of EVs encompasses enzymes, signaling proteins, transcription factors and surface receptors [7,27,28]. The entire spectrum of these bioactive molecules can be delivered to the recipient cells and modulate their biological function. The ability of EVs to encapsulate their cargo within lipid bilayers and protect it from degradation enzymes renders EVs valuable biomarkers. Moreover, EVs isolated from pathological tissues may have tissue-specific surface markers, thus facilitating increased specificity as diagnostic biomarkers. Additionally, miRNAs derived from EVs probably reflect disease-specific processes, posing an enhanced specificity of the particular molecular markers [29,30,31].

**Figure 1:**
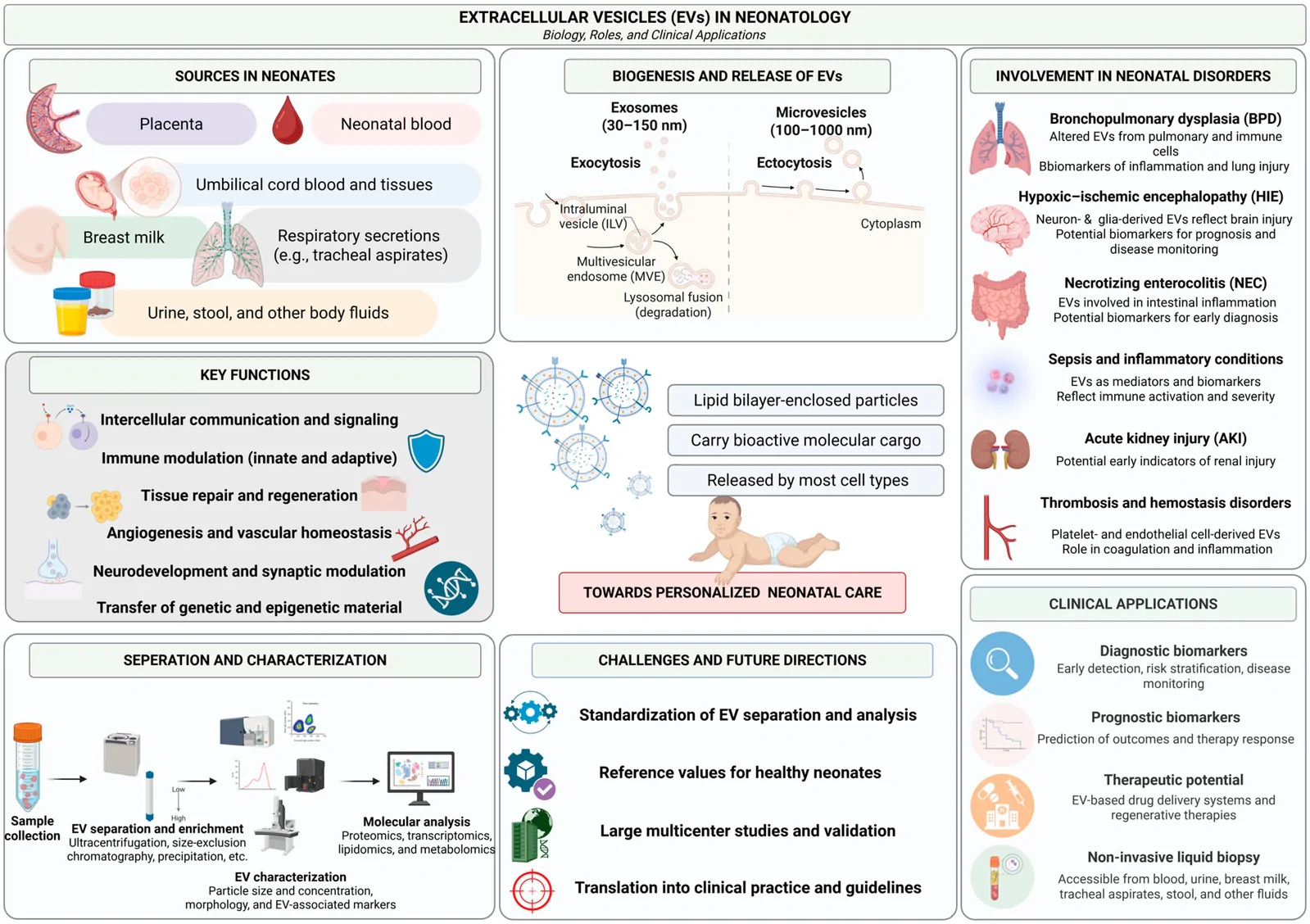
Overview of extracellular vesicles in neonatal health and disease. The figure summarizes the major neonatal sources and biological functions of extracellular vesicles (EVs), their principal biogenesis and release pathways, and their potential involvement in neonatal disorders, including bronchopulmonary dysplasia, hypoxic–ischemic encephalopathy, necrotizing enterocolitis, sepsis, acute kidney injury, and thrombotic or hemostatic disorders. Common EV separation, enrichment, characterization, and molecular analysis approaches are also illustrated. Extracellular vesicles (EVs) are increasingly investigated as diagnostic and prognostic biomarkers, components of non-invasive liquid biopsy, and potential therapeutic tools. Key challenges include methodological standardization, establishment of neonatal reference ranges, validation in large multicenter studies, and translation into clinical practice. Created in BioRender. Fortis, S. (2026) https://BioRender.com/vgi6d67.

Extracellular vesicles (EVs) are present in various body fluids, such as peripheral and umbilical cord blood, urine, tears, saliva, tracheal aspirates and breast milk. Moreover, laboratory methods enable the purification and enrichment of EVs from these body fluids, making the detection of EVs and miRNA feasible, even if the initial concentration is too low. Finally, EVs and their content reflect the status of the cell of origin in normal and pathological states; thus, the non-invasive sampling of biological fluids can substitute for tissue biopsies and EVs can serve as liquid biopsies. All these characteristics, taken into consideration, indicate that EVs are valuable in understanding disease pathophysiology, as well as diagnostic–prognostic biomarkers and potential therapeutic targets [32]. Despite the increasing literature about EVs during the neonatal period, the comprehension of their role necessitates a broad approach, which extends beyond the simple documentation of the available studies. In the present narrative review, we attempt to incorporate the existing data into a uniform framework, interconnecting the developmental changes in EVs during the transition from intrauterine to extrauterine life with their involvement in the pathophysiology of the main neonatal disorders. Simultaneously, we assess their potential utilization as diagnostic and prognostic biomarkers, while we analyze the rising preclinical data about their usefulness as therapeutic tools. Thus, the current review aims to connect the developmental biology of EVs with the pathophysiology, their value as biomarkers, as well as the prospect of therapeutic translation in neonatology. Although there is growing evidence regarding the implication of EVs in neonatal conditions, both as diagnostic biomarkers and therapeutic tools, the current review tends to summarize and comprehensively incorporate the existing data, towards a better understanding of their utility and possible clinical application. An overview of EVs in neonatal health and disease is presented in Figure 1.

## 2. Methods

In order to conduct an updated and detailed narrative review on the implications of EVs in neonatal health and disease, a thorough search of the available literature has been performed. PubMed- and Scopus-indexed manuscripts were mainly included, while the language was restricted to English. Original research studies and relevant review articles were considered, with particular emphasis on recent publications and studies directly relevant to neonatal physiology, disease, biomarker applications, and emerging therapeutic approaches. Complementary studies in adults have also been taken into consideration in order to thoroughly outline the multiple functions and properties of EVs. Great attention has been paid to the incorporation of the most recent and updated publications.

For the purposes of the current review, we use the generic term “extracellular vesicles” (EVs), in accordance with the MISEV2023 recommendations of the International Society for Extracellular Vesicles (ISEV) [8]. Biogenesis-related terms such as “exosomes” or “microvesicles” are used only when the respective origin has been adequately demonstrated. When discussing individual studies in which such characterization was not established, the terminology originally used by the study authors is reported for accuracy, while recognizing that these terms may not fully conform to current MISEV2023 nomenclature.

## 3. The Role of Extracellular Vesicles as Diagnostic Biomarkers in Neonatal Disorders

### 3.1. Extracellular Vesicle Profiles Across Gestational Age and Their Potential as Biomarkers of Preterm Birth

The limits of viability are consistently decreasing and nowadays are set at 22 weeks of gestation in many developed countries, while resuscitation and active care are offered even at 21 weeks’ gestational age in a particular center [33]. The ascertainment of novel biomarkers is of increasing concern, in order to better comprehend the pathophysiology, diagnosis and management of prematurity-related diseases. Increasing awareness about the multiple roles of EVs has yielded a novel identification of their involvement in feto-maternal communication and signaling [34].

The EVENT study (Extracellular Vesicles in Early preterm Neonates and Thrombin generation) focused on the characterization of circulating EVs in preterm neonates during the perinatal adaptation period in comparison to full-term controls [35]. A total of 101 premature neonates of 24^+0^ to 30^+6^ weeks’ gestational age were recruited in the study and umbilical cord or peripheral blood samples were subsequently collected at birth and during the first 2 weeks of age, while 66 full-term neonates served as controls and were sampled at any time during the first 4 days of life. Evolving alterations with regard to levels, size and cellular origin of EVs were observed in preterm neonates, mostly due to an increase in platelet, TF and endothelial EVs, while a concomitant differentiation in EV proteomic profile was demonstrated. Dynamic EV changes were also detected in the term group, although to a lesser extent. The findings indicate the high incorporation of EVs in neonatal hemostasis, immunity and pulmonary pathophysiology, while the dynamic EV profile in relation to prematurity may be attributed to extrauterine maladaptation or the onset of inflammatory conditions in this group [35].

Ohta et al. investigated the physiology of exosomes in association with prematurity by measuring the concentration of serum-derived exosomes in preterm neonates at birth and successively throughout the first year of life [36]. The concentrations of exosomes increased gradually during the first year of life, probably due to the involvement of exosomes in organ development and maturation. A positive correlation was detected between gestational age, multiple births and exosome levels, presumably reflecting the immaturity of preterm neonates and the perinatal maladaptation in twin pregnancies. Respiratory disease had no impact on exosome concentrations [36].

Additionally, Bruschi et al. compared the proteome of microvesicles derived from cultured Mesenchymal Stem Cells (MSCs) from umbilical cord blood of preterm (28–32 weeks of gestation) and term neonates [37]. A distinctive proteomic profile was demonstrated between the two groups, mostly regarding augmented inflammatory and oxidative metabolic pathways in the preterm group. The results confirm the well-established link between inflammation and preterm delivery, suggesting that microvesicles may serve as biomarkers of this strong correlation [37].

Despite the distinct EV patterns in preterm and full-term neonates, the greatest benefit would be offered by the application of EVs as biomarkers in the risk stratification of preterm delivery. Amniotic fluid-derived and maternal plasma-derived exosomes demonstrated distinctive proteomic profiles in relation to preterm delivery and premature prolonged rupture of membranes, chiefly associated with inflammatory and metabolic signaling, denoting the potential application of exosomes as biomarkers in pregnancy complications [38,39]. On the contrary, another study demonstrated that umbilical cord blood-derived exosomes had no association with specific underlying biological function and their value as diagnostic biomarkers was shown to be limited [40].

Interestingly, Cantonwine et al. identified a unique microparticle proteomic profile, which was expressed as early as 10–12 weeks of gestation and was linked to spontaneous preterm birth at ≤34 weeks of gestation [41]. Microparticle-derived biomarkers were mainly associated with coagulation, inflammatory and immune-modulatory processes, already evident since the end of the first trimester of pregnancy, indicating the early origin of many complications of parturition [41]. Similarly, McElrath et al. identified a set of circulating microparticle proteins tested at 10–12 weeks of gestation that potentially can be used for risk stratification of spontaneous preterm delivery ≤ 35 weeks. The finding may be especially useful for nulliparas, where clinical factors, such as history of prior preterm delivery are lacking [42]. Zhao et al. proposed microvesicle phosphatidylserine (34:0), assessed between 12 and 24 weeks of gestation, as another potential biomarker of preterm delivery [43].

Moreover, Menon et al. determined placental EVs and their protein cargo in maternal plasma across all trimesters of pregnancy, both in term and spontaneous preterm birth, and suggested their role as a “mirror” of the functional status of the placenta [34]. Lower levels of placental EVs were measured in the preterm group and a differential expression of the EV proteome was also demonstrated. Placental EV protein cargo in term pregnancies was associated with epithelial–mesenchymal transition signaling pathways, while the preterm group demonstrated downregulation of coagulation and complement activation. Intriguingly, nonspecific inflammatory pathways were upregulated in both groups, but first-trimester preterm protein cargo had more inflammatory markers in comparison with the term group. This particular finding indicates that an inflammatory process is more intense in early stages of preterm labor, associated with tissue remodeling, although a quantitative and functional analysis of differentially expressed proteins was not performed [34]. The same research group investigated the exosomal miRNA in maternal plasma throughout pregnancy and at birth in term and preterm births and identified a unique miRNA profile in each group, chiefly associated with Transforming Growth Factor β (TGF-β) signaling, p53 and glucocorticoid receptor signaling [44].

Furthermore, placenta-derived exosomes were measured at significantly lower levels in Small for Gestational Age (SGA) and Fetal Growth Restriction (FGR) cases in comparison to controls, in maternal and fetal plasma. The decline was more pronounced according to the severity of the disease, indicating the potential utility of placental exosomes as a biomarker of placental insufficiency and fetal growth status [21].

Conclusively, EVs can be used as a valuable and non-invasive indicator of the maternal–fetal interface, as well as of the intrauterine environment, which are both physically and ethically difficult to access. However, whether the observed EV alterations primarily reflect physiological developmental trajectories or early signatures of prematurity-related pathology remains to be clarified. The available studies on EV profiles in relation to gestational age are summarized in Table 1.

### 3.2. The Role of Extracellular Vesicles (EVs) as Diagnostic Biomarkers of the Neonatal Hemostatic Mechanism

The procoagulant activity of EVs is mainly ascribed to phosphatidylserine (PS) expression by them, which represents a negatively charged phospholipid that interacts with gamma-carboxyglutamic acid of coagulation factors, triggering the formation of tenase and prothrombinase complexes. The interaction of red blood cell-derived EVs with the anticoagulant protein S (PS), as well as their involvement in fibrinolysis due to the presence of plasminogen on their surface, have also been demonstrated, although not thoroughly investigated [45]. Furthermore, EVs derived from endothelial cells and monocytes expose TF [46], a phenomenon also evident in EVs released by tumor cells, contributing to the procoagulant propensity of cancer patients [47,48]. Nevertheless, recent evidence indicates that the procoagulant activity of EVs is more complex, involving cellular components leading to expansion of coagulation surfaces and intercellular interaction at sites of vascular injury [17].

Platelet-derived EVs (PDEVs) are released by platelets during activation or apoptosis and represent a significant procoagulant effector, besides their role in immune modulation, angiogenesis and cellular repair mechanisms [49,50]. Furthermore, PDEVs constitute the major fraction of EVs in the blood (60–90%) [51]. Michelson et al. evaluated the procoagulant activity of PDEVs in preterm and term neonates, as well as adults. A significant increase in PEDVs (CD42b) after activation was demonstrated both in term and preterm neonates in comparison to adults. However, the procoagulant activity of PEDVs after activation was diminished in preterm neonates compared with term newborns and adults, due to relatively diminished Factor V (FV) levels, probably contributing to a bleeding tendency in preterm neonates [52]. Similarly, Schmugge et al. demonstrated higher levels of PEDVs in neonates compared with adults, as well as enhanced activation of neonatal platelets and a link between von Willebrand Factor (vWF) binding and platelet activation, probably due to a higher percentage of large vWF multimers in neonatal plasma [53]. A comparative study on PEDV levels in term and preterm newborns indicated significantly higher levels in the preterm group, which may reflect a possible compensatory mechanism in neonatal hemostasis [54]. Sequential measurements of EVs in preterm neonates on day of life (DOL) 1 and 3 showed increased levels of both small and large EVs on DOL 3 compared to DOL 1, but a reduction in PEDVs in the same time period was demonstrated, suggesting an early platelet activation after preterm delivery [55].

Additional studies on neonatal EVs demonstrated no difference or slightly elevated levels of EVs in umbilical cord blood compared with adults; however, neonatal EVs presented significantly enhanced procoagulant activity and a substantial functional impact on the neonatal coagulation process [56,57]. Similarly, Uszyński assessed the procoagulant activity of microparticles, TF and Tissue Factor Pathway Inhibitor (TFPI) in umbilical cord blood and matching maternal blood. Levels of microparticles and TF were higher, while TFPI concentration was lower in cord plasma rather than maternal, indicating a procoagulant compensatory mechanism in fetal blood and a deviation in fetal and maternal microparticle release and clearance [58]. The procoagulant activity of neonatal EVs was also verified by Korbal et al., who measured increased levels of TF bearing EVs in a preterm group compared with a term one. A negative correlation of TF bearing EVs with gestational age and birthweight was shown, suggesting a prothrombotic tendency in preterm newborns [59]. On the contrary, a reduced procoagulant activity of neonatal EVs in comparison to older children and adults was found by Karlaftis et al. [60].

Additional studies have shown no differences between term and preterm neonates regarding PDEVs (CD41+CD36+), activated platelet EVs (CD41+CD62+), or endothelial EVs (CD31+CD105+) in umbilical cord blood. Nonetheless, particular surface markers, such as platelet (CD36), platelet activation (CD62) and endothelial (CD31) markers, were less expressed in the preterm group, reflecting the developmental aspects of neonatal hemostasis [61,62].

Finally, a qualitative and quantitative study of exosomes in neonates compared with adults demonstrated significantly smaller size of neonatal exosomes, as well as a distinct protein cargo of them [63]. Specific proteins were expressed at increased levels in neonates, mainly proteins associated with activation of platelets, granule secretion and coagulation, such as Factor VIII (FVIII) and vWF, in accordance with the higher levels of these coagulation factors in neonates [64]. Similarly, the concentration of the anticoagulant PS was measured at lower levels in neonatal exosomes compared with adults, as in neonatal plasma too. The under-expressed proteins in neonatal exosomes were mostly immunoglobulins, in line with the relative immunodeficiency of neonates [63,65].

Uniformly, all these cumulative data indicate that EVs are major orchestrators of the well-balanced and fine-tuned neonatal hemostatic mechanism. Heretofore, research evidence indicates that EVs can serve as biomarkers of platelet activation and maturation, procoagulant surface exposure and endothelial status in neonates, as well as biomarkers of prothrombotic and hemorrhagic risk in this age group. Their potential utilization as diagnostic biomarkers either as standalone methods or in conjunction with conventional and newer viscoelastic coagulation tests should be explored in future studies, as well as their possible therapeutic usefulness. The data indicating the role of EVs as diagnostic biomarkers of neonatal hemostasis are presented in Table 2.

### 3.3. The Utility of Extracellular Vesicles (EVs) as Diagnostic Biomarkers of Neonatal Respiratory Disorders

Respiratory diseases of the newborn are probably the commonest clinical problems encountered in everyday practice of neonatology, with substantial impact on short-term and long-term mortality, neurodevelopmental outcome and life-long quality of life. Bronchopulmonary dysplasia (BPD) represents a heterogeneous spectrum of lung growth and alveolarization disruption, clearly marked by a prolonged requirement of respiratory support [66]. Mounting evidence indicates a key role of EVs in the pathophysiology of BPD.

First of all, exosomes from umbilical cord blood of preterm neonates with BPD have been shown to exert a negative effect on endothelial angiogenesis, cell proliferation and capillary tube formation in vitro in comparison with preterm neonates without BPD [67]. Additionally, EVs demonstrate a novel link in the well-known lung–brain axis of preterm newborns. Plasma EVs from preterm neonates at high risk for BPD (need for high fraction of inspired oxygen, FiO_2_ ≥ 30%) expressed particular inflammatory markers compared with preterm neonates at low risk for BPD (FiO_2_ < 30%). The adoptive transfer of the EVs from the high O_2_ group in healthy newborn mice resulted in increased lung inflammation, impaired alveolarization and vascular maldevelopment, the pioneer characteristics of BPD. Moreover, EVs crossed the blood–brain barrier (BBB) of newborn mice and induced brain injury, such as inflammation and cell death in the hippocampus [68].

Regarding the role of EVs as BPD biomarkers, Go et al. explored the incorporation of EV miRNAs in the pathogenesis of BPD in preterm neonates, as well as in a mouse model of the disease, demonstrating a distinct miRNAs profile in BPD compared with non-BPD newborns, also verified in the mouse model of the disease [69]. MiR-21, a key regulator in deranged repair, inflammation and fibrosis of the developing lung, was also upregulated in BPD patients on DOL28 compared with DOL0, exerting a good diagnostic performance (AUC 0.85) [69].

Another potential biomarker of BPD was demonstrated by Lal et al. and refers to miR-876-3p [70]. In particular, the research team measured higher concentrations of exosomes in tracheal aspirates of neonates with BPD compared with gestational age-matched full-term controls, though a distinct pattern with smaller-sized exosomes was found in the patient group. Furthermore, a specific miRNA profile was demonstrated at birth in Extremely Very Low Birthweight (ELBW) neonates who later developed BPD, compared with those that did not. Diminished exosomal miR-876-3p levels at birth were associated with the highest prognostic power of severe BPD and were also demonstrated in established BPD [70].

More recently, Ransom et al. collected tracheal aspirate samples from preterm neonates, either in the late canalicular stage (gestational age of 22^+0^–26^+6^ weeks) or saccular stage of lung development (gestational age of 27^+0^–34^+6^ weeks) [71]. No difference regarding the concentration of EVs was demonstrated between the two groups. However, EVs of larger mean and median diameter were found in the late canalicular stage compared with the saccular stage of lung development. A variety of EV markers were detected, such as tetraspanins, epithelial markers (CD326, CD133, CD142 and ROR1) and markers expressed by immune cells, for example, CD3 and CD40. Higher levels of CD^+24^ EVs and a marginally elevated concentration of CD^+14^ EVs were found in the late canalicular stage compared with the saccular stage of lung development and were associated with the later development of BPD [71]. CD24 is highly expressed by the epithelium of the respiratory tract and by lung macrophages in adults [72]. Moreover, CD24 represents a well-known inhibitor of the nuclear factor kappa-light-chain-enhancer of activated B cells (NF-kB) pathway, which regulates lung repair and alveolar development. Excessive or insufficient NF-kB signaling is associated with BPD development and impaired alveologenesis, respectively [71,73,74].

Finally, Amatya et al. assessed the plasma-derived EV proteome as a predictor of BPD development and identified a unique signature of nine EV proteins associated with a good predictive performance for BPD, particularly HNRNPM [75]. These proteins are functionally involved in mRNA splicing, RNA metabolism and inflammation. Dysregulation of Vascular Endothelial Growth Factor A (VEGFA) alternative splicing may lead to maldevelopment of aerocytes, a type of endothelial cell critical for adaptation of the lung to gas exchange. Deranged development of aerocytes may take part in BPD evolution [76,77].

All these findings taken into consideration indicate the potential utility of EVs as liquid biopsies of the respiratory tract and early biomarkers of BPD, although their specificity for BPD pathophysiology over developmental maturation and concurrent inflammatory processes remains to be established. The available data on EVs role as diagnostic biomarkers of neonatal respiratory disorders are presented in Table 3.

### 3.4. The Implication of Extracellular Vesicles (EVs) as Diagnostic Biomarkers of Central Nervous System Disorders in the Newborn

Early biomarkers and reliable predictors of Central Nervous System (CNS) disorders could lead to prompt identification of brain injury, aggressive preventive and therapeutic interventions and EVs could be a useful tool in this direction. First of all, EVs are able to cross the ΒΒΒ bilaterally from brain tissue to the bloodstream and vice versa, implementing a significant cross-talk between CNS and peripheral circulation both in health and disease [78]. Moreover, recent evidence indicates that EVs are involved in intercellular communication between neurons, oligodendrocytes, glial cells and astrocytes. The modification of signal transduction by EVs leads to alteration in neurogenesis, gliogenesis, synaptogenesis, network circuit formation, synaptic pruning and myelination. The potential role of EVs as disease biomarkers has been recently suggested in Down syndrome, as well as fetal alcohol syndrome [79].

Intraventricular hemorrhage (IVH) is a major CNS complication of prematurity, inversely correlated to gestational age and attributed to the fragility of the germinal matrix in preterm neonates, which can progress to post-hemorrhagic ventricular dilatation and hydrocephalus [80]. Spaull et al. analyzed the EV pattern in cerebrospinal fluid (CSF) of three extremely preterm neonates with post-hemorrhagic hydrocephalus (PPH) and identified a particular exosome profile and a distinct cargo of brain-related mi-RNAs in them. Additionally, a progressive decrease in EV concentration was demonstrated over time in one out of three patients, while miR-1911-5p levels, a brain-specific miRNA, were increased [81].

Furthermore, hypoxic–ischemic encephalopathy (HIE) is another major CNS complication, mainly in full-term neonates, caused by perinatal cerebral ischemia and oxygen delivery failure, which, nowadays, is managed with therapeutic hypothermia. Nevertheless, HIE is still associated with long-term neurodevelopmental disability; thus, the identification of disease-specific biomarkers and early predictors of outcome is of paramount importance [82]. Interestingly, Goetzl et al. investigated the role of neuronal exosomes isolated from peripheral blood samples of neonates with HIE under therapeutic hypothermia at sequential timepoints and quantified their cargo, particularly synaptopodin (SYNPO), synaptophysin (SYN), neuron-specific enolase (NSE) and mitochondrial cytochrome c oxidase (COX IV) [83]. The intriguing finding was that the neutral or decreasing slope of SYNPO was correlated with extended length of stay, discharge on seizure medication and composite neuroimaging score, concluding that neuronal exosomes and their cargo may serve as a “troponin-like” test in neonatal acute brain injury [83]. The same research group extended their sample size and identified that decreasing synaptopodin levels and increasing lipocalin-2 (NGAL) levels were correlated with the MRI injury score [84].

Additionally, Marell et al. have explored the possible usefulness of EVs as molecular markers of neonatal brain iron status [85]. The investigators measured the concentrations of exosomal Contactin-2 (CNTN2) and brain-derived neurotrophic factor (BDNF) in neonatal blood circulation, in conjunction with the iron status. Both of these factors, CNTN2 and BDNF, demonstrate neuronal expression and are implicated in neurodevelopment. A positive correlation was observed between CNTN2 and ferritin levels, while a negative relationship was shown between BDNF and ferritin levels [85].

Finally, Tan et al. examined the protein content of EVs in CSF of neonates with Acute Bilirubin Encephalopathy (ABE) in comparison with age-matched controls [86]. Certain proteomic alterations of CSF EVs were demonstrated in the patient group compared with the healthy controls, mostly associated with the immune–inflammatory response [86].

Despite these promising findings, evidence remains preliminary and is derived from small and heterogeneous neonatal cohorts; therefore, the diagnostic and prognostic performance of EV-derived CNS biomarkers requires validation in larger prospective studies.

### 3.5. The Position of Extracellular Vesicles (EVs) as Diagnostic Biomarkers of Necrotizing Enterocolitis (NEC)

Necrotizing enterocolitis (NEC) represents the most critical gastrointestinal tract complication of preterm neonates, inversely related to gestational age. The underlying pathophysiologic mechanisms include intestinal ischemia–hypoxia, metabolic derangement, enhanced inflammatory response and platelet activation, and, finally, advancing to multi-organ dysfunction [87,88]. The discovery and clinical application of disease-specific diagnostic biomarkers would aid in the timely diagnosis of NEC, as well as its differential diagnosis from neonatal sepsis, whose early symptomatology usually overlaps. Galley et al. investigated the role of urine EV-derived miRNA in premature infants ≤ 34 weeks of gestation with medical NEC (MNEC, not requiring surgery), surgical NEC (SNEC), non-NEC sepsis and healthy age-matched controls, at sequential timepoints [89]. Urine samples were rich sources of EVs and sample eluents were positive for Alix and CD63 EVs. The EV-miRNA cargo was differentially expressed between the study groups, particularly SNEC vs. non-NEC sepsis (including miR-376a-3p, miR-31–5p, and miR-518a-3p), MNEC vs. controls (including miR-604), SNEC vs. controls, as well as MNEC and SNEC vs. controls (including miR-5186, miR-139–3p, miR-5703 and miR-604). No differences in miRNAs expressed was observed in the MNEC vs. SNEC group. The differentially expressed miRNAs were mostly linked to inflammation-associated molecules. Unfortunately, there was no early discrimination between the NEC and no-NEC sepsis groups. The alterations were observed 6–14 days after the disease onset, when the most critical clinical window has been lost. Thus, although urine-derived EV miRNAs appear to be biologically informative, their clinical utility as early diagnostic biomarkers of NEC remains to be established [89].

### 3.6. The Role of Extracellular Vesicles (EVs) as Diagnostic Biomarkers of Retinopathy of Prematurity (ROP)

Extracellular vesicles (EVs) are present in several body fluids, including tears. The EV signature of tears was recently analyzed in correlation with retinopathy of prematurity (ROP) development [90]. The study included 33 preterm neonates who developed ROP and 14 preterm newborns without ROP who served as controls. A total of 18 neonates in the ROP group required treatment, while 15 did not. The tear sample was collected at 34 weeks postmenstrual age; then, EVs were appropriately isolated and quantitative real-time PCR was performed to analyze the miRNA panel of EVs. Regarding the EVs characterization, a high surface expression of CD9 was demonstrated in the ROP group, while low expression of epithelial cell adhesion molecule (EpCAM) and DC147 co-existed. Regarding miRNA expression, 202 miRNAs were differentially expressed in the ROP group compared with the no-ROP group. Differential expression of miRNAs was also identified in treatment-requiring ROP cases vs. non-treatment-requiring ROP cases. Among the differentially expressed miRNAs, miR-520a-5p demonstrated strong diagnostic accuracy and diagnostic efficacy in discriminating treatment-requiring ROP cases. Moreover, a significant dose–response relationship between miR-520a-5p and ROP severity was demonstrated among all study groups. However, levels of miR-520a-5p were also negatively correlated with gestational age and its impact was directly related to gestational age. Notably, miR-520a-5p is tightly incorporated in a network of multiple angiogenic and inflammatory factors, such as VEGF and NF-kB. A potential advantage of ocular EVs may be that they reflect more accurately the local disease status compared with plasma or serum biomarkers [90].

The available studies on the utility of EVs as diagnostic biomarkers of CNS disorders, NEC and ROP are summarized in Table 4.

### 3.7. Extracellular Vesicles (EVs) as Diagnostic Biomarkers of Endothelial Dysfunction in Neonates

The endothelium represents the monolayer of cells lining the internal vessel wall and exerting pivotal functional roles, not only as a barrier, but also in maintaining circulatory homeostasis, vascular tone, coagulation and angiogenesis control. Extracellular vesicles (EVs) have emerged as favorable biomarkers of endothelial status and cardiovascular disease [91].

First of all, elevated levels of endothelial microparticles have been identified in umbilical cord venous blood from low birth weight neonates, although the study results were not officially published [92]. Similarly, Simoncini et al. evaluated the role of endothelial microparticles in term and preterm neonates and identified a unique pattern in the preterm group, characterized by increased levels of endothelial microparticles and induction of endothelial senescence by them [93]. Additionally, endothelial microvesicles have been assessed in neonates on Extracorporeal Membrane Oxygenation (ECHMO) and increased levels of total and endothelium-specific microvesicles were identified in the patient group compared with healthy controls. Endothelial microvesicles also demonstrated a positive correlation with endothelial and inflammatory markers, as well as clinical severity, suggesting their potential utility as biomarkers of endothelial activation and injury [94]. Nevertheless, the influence of the underlying clinical condition and the impact of the extracorporeal circuit on EV release should not be ignored [46].

Furthermore, endothelial microparticles have been evaluated in neonatal hemolysis due to ABO incompatibility as a marker of endothelial injury. Besides erythrocytes, ABO antigens are expressed on several tissue surfaces including endothelium. Endothelial microparticles were measured at increased concentrations in ABO hemolytic disease in comparison to Rh hemolytic disease and healthy controls, and the levels were correlated to severity of hyperbilirubinemia. Endothelial microparticle concentrations declined substantially after intervention, such as phototherapy and especially exchange transfusion. The study underscores the IgG-mediated endothelial damage in cases of ABO hemolytic disease, similar to IgG-mediated erythrocyte destruction and implies a possible long-term effect on vascular status. Endothelial microparticles could be employed as surrogate markers of disease severity [95]. Zhu et al. likewise explored the role of endothelial microparticles in hemolytic disease of the newborn and revealed higher levels of them in ABO hemolytic disease compared with Rh hemolytic disease and healthy controls [96]. A similar study evaluated the microparticle pattern in neonates with non-hemolytic hyperbilirubinemia in comparison with healthy controls and identified significantly higher levels of total apoptotic and endothelial-derived apoptotic microparticles in the patient group compared with the control group, before and after phototherapy [97]. Collectively, these findings suggest that endothelial EVs may reflect vascular injury in neonates; however, their disease specificity remains uncertain, as EV release may represent a common response to inflammation, hemolysis, extracorporeal circulation, or other forms of endothelial stress. A synopsis of the related studies is presented in Table 5.

### 3.8. The Utility of Maternal and Placental Extracellular Vesicles (EVs) as Diagnostic Biomarkers of Preeclampsia

Preeclampsia is a significant pregnancy-related disease, posing an increased risk both for the mother and the fetus and several studies have explored the possible alterations of the EV profile in preeclamptic pregnancies [98]. Initially, Campello et al. demonstrated an enhanced procoagulant activity of EVs in pregnancies complicated by preeclampsia in comparison to normotensive pregnancies [99]. Interestingly, total microparticles (annexin+), activated platelet-derived (P-selectin+), leukocyte-derived and TF bearing microparticles were measured at significantly increased levels in umbilical cord and peripheral blood in preeclamptic women compared with normotensive women. Platelet-derived microparticles (CD61+) were measured at lower levels in preeclamptic women rather than controls, probably alongside the reduced platelet count. The discrepancies between the two groups were more pronounced in cord venous blood compared with peripheral venous blood, suggesting that microparticles are powerful procoagulants present in abundance in fetal circulation, counter-balancing the immature hemostatic mechanism of the fetus [99].

Jia et al. have detected a differential EV proteomic signature in umbilical cord blood in preeclamptic vs. normotensive pregnancies, suggesting a pathophysiologic involvement of EVs in preeclampsia, probably through the complement and coagulation cascade [100]. Similarly, Xueya et al. identified that exosomal miR-125a-5p in preeclampsia downregulated angiogenesis by suppressing VEGFA expression in the placenta, directly related to the pathogenesis of preeclampsia [101].

Extracellular vesicles (EVs) arise as strong indicators of feto-maternal communication and interaction; however, more data are necessary to validate and expand their role in pregnancy and fetal complications.

### 3.9. Miscellaneous Applications of EVs as Diagnostic Biomarkers

The EVs applications constitute a rapidly expanding field. Kunte et al. investigated the EV profile in mother–neonatal pairs of lean and adipose neonates and identified a differential expression of adipocyte-derived small EVs (ADsEVs) miRNA in maternal and cord blood plasma samples at birth between lean and adipose neonates [102]. The differentially expressed miRNAs were involved in adipogenesis-related pathways. Particular maternal ADsEVs miRNAs are associated with adipose dysfunction, suggesting a signaling role for them in fetal and neonatal adiposity [102].

Interestingly, Alhamdan et al. explored the involvement of EVs in postoperative organ dysfunction in neonates and infants with congenital heart disease. An organ dysfunction (OD) and a non-organ dysfunction (NOD) group were included, which were both exposed to cardiopulmonary bypass (CPB). Peripheral blood (plasma) samples were collected at four distinct timepoints: at baseline, in the rewarming phase during CPB, upon Intensive Care Unit (ICU) admission and on postoperative day 1 [103]. The EVs miRNA signature in the OD group was mainly associated with upregulated apoptosis, proinflammatory pathways and neutrophil extracellular trap (NET) formation across all timepoints, while complement and coagulation pathways were downregulated towards the end of surgery [103]. The dynamic alteration of EV cargo at sequential timepoints is very interesting, as it suggests that EVs may act as dynamic biomarkers, depicting in real time the neonate’s evolving biological response to a significant surgery and inflammatory stress.

The EV profile as biomarkers in preeclampsia, neonatal adiposity and postoperative OD is presented in Table 6.

## 4. The Role of Extracellular Vesicles as Therapeutic Tools in Neonatal Disorders

The therapeutic properties of Extracellular Vesicles derived from the secretome (conditioned medium) of stem/progenitor cells as regenerative, immunomodulatory and anti-inflammatory effectors, as well as drug delivery mediators, are gaining increasing evidence and bode well as breakthrough therapeutic options [104]. Nevertheless, the data are still experimental and are yielded mostly from preclinical studies. The most potent therapeutic agents are Mesenchymal Stem/Stromal Cells Extracellular Vesicles (MSC-EVs). Stem cells can differentiate into multiple cell types and represent a prominent and reinforced repair mechanism. Stem cells are divided into two main types: embryonic and adult stem cells. Embryonic stem cells originate from the inner cell mass of a blastocyst and are pluripotent cells, capable of differentiating into any cell type, as well as self-renewing. These inherent properties are partially attributed to stem cell-derived EVs. Adult stem cells also release EVs that can modify other cells in the local or distant environment [7]. The therapeutic application of EVs released by stem and progenitor cells overcomes many obstacles related to cell therapy. Extracellular vesicle-based therapeutics are a cell-free treatment, lacking the risks of live cell administration, such as microvascular occlusion, infusion toxicity and ectopic tissue formation. Moreover, EVs exhibit greater and long-term stability, thus enabling easy and low-cost storage [105]. Extracellular vesicles (EVs) from allogeneic donors demonstrate a sufficient safety profile with low immunogenicity risk preclinically; thus, the necessity of autologous sources is bypassed. Additionally, EVs can be loaded with a variety of therapeutic cargo, targeted to specific tissues, thus minimizing off-target effects. Finally, therapeutic EVs lack the concern of tumorigenesis associated with MSC-based therapies, while EVs are biologically inert and their biological function will not be altered by the in vivo environment [106,107].

**Figure 2:**
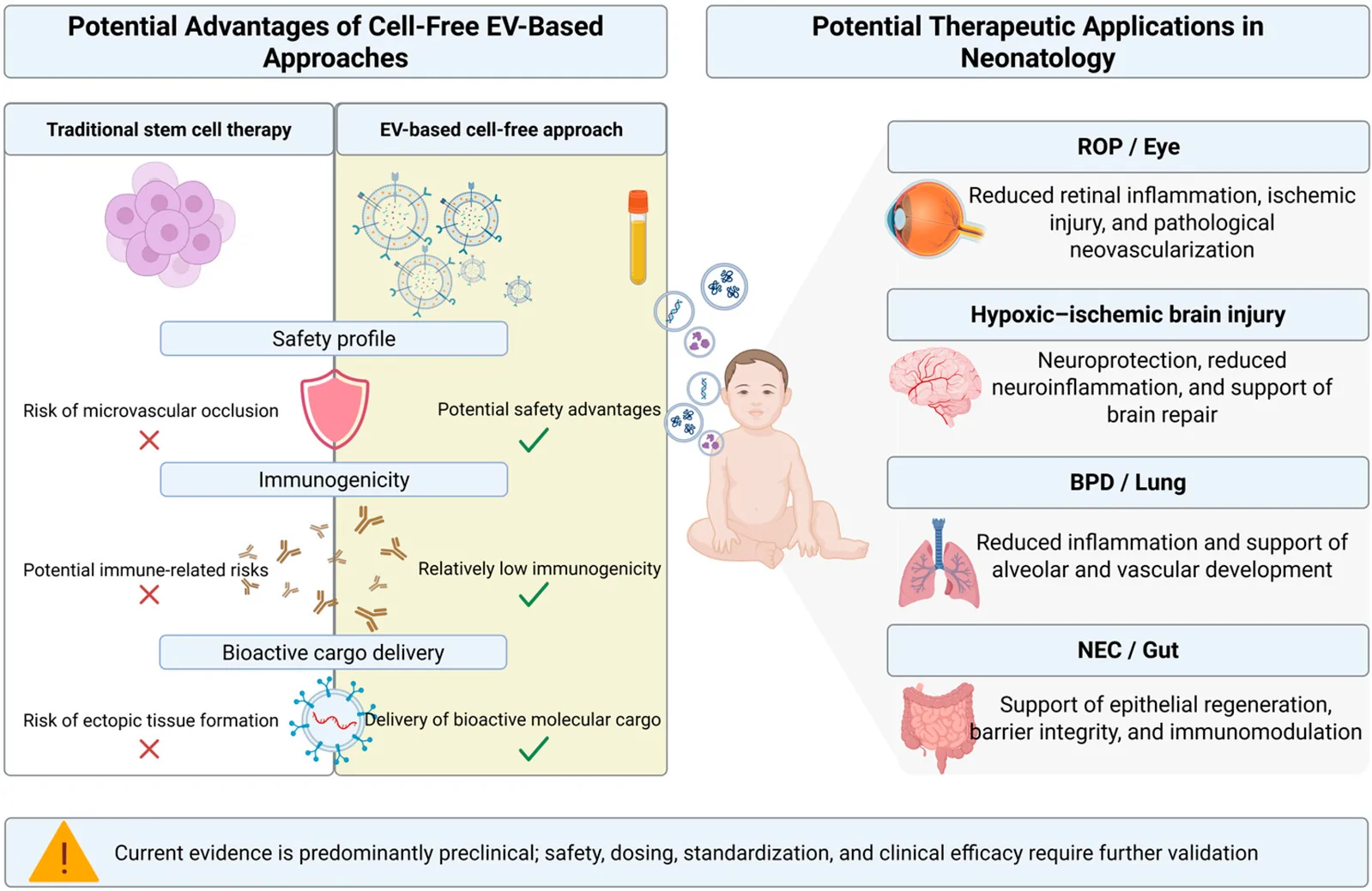
Potential advantages and emerging therapeutic applications of extracellular vesicle-based, cell-free approaches in neonatology. Compared with traditional stem cell therapy, EV-based approaches may offer potential safety advantages, relatively low immunogenicity, and the capacity to deliver bioactive molecular cargo, while avoiding certain risks associated with live-cell administration, such as microvascular occlusion and ectopic tissue formation. Preclinical studies suggest potential therapeutic effects in retinopathy of prematurity (ROP), hypoxic–ischemic brain injury, bronchopulmonary dysplasia (BPD), and necrotizing enterocolitis (NEC), mediated through anti-inflammatory, neuroprotective, pro-regenerative, and vascular-modulating mechanisms. Current evidence remains predominantly preclinical; therefore, safety, optimal dosing, production standardization, biodistribution, and clinical efficacy require further investigation and validation. Created in BioRender. Fortis, S. (2026) https://BioRender.com/vgi6d67.

With regard to neonatology, the potential therapeutic usage of EVs has been explored in various neonatal disorders, such as BPD, NEC and ROP, and will be subsequently elaborated. An overview of the potential advantages and emerging therapeutic applications of EVs in neonatology is presented in Figure 2, while the available studies are summarized in Table 7.

### 4.1. The Therapeutic Utility of EVs in BPD

As aforementioned, BPD is a leading cause of preterm morbidity and mortality, posing an urgent need for new and definitive therapeutic options. The main aspects of EVs’ protective role in BPD development involve anti-inflammatory processes, pro-angiogenesis, alteration of lung macrophage phenotype, regulation of cell proliferation, migration and differentiation, as well as control of mitochondrial activity and oxidative stress [115,158]. The surface membrane proteins and the protein and miRNA cargo activate signal transduction in target cells and modulate the biological function of target cells [158]. Moreover, EVs are physically entrapped in pulmonary capillaries; thus, they accumulate and remain in the lungs, particularly large EVs (>200 nm) [159].

First of all, EVs released by bone marrow-derived mesenchymal stem cells (BM-MSCs) and Wharton’s jelly MSCs (WJ-MSCs) have been shown to exert multiple beneficial effects in mouse models of BPD, such as reduced pulmonary macrophage influx, suppressed pulmonary vascular remodeling, enhanced alveolarization and lung angiogenesis, as well as ameliorated pulmonary hypertension [108,109]. An intriguing finding is that EVs localize in the lung and exert anti-inflammatory and immunosuppressive actions, leading to restoration of alveolar architecture, downregulation of fibrosis and vascular remodeling, as well as enhanced exercise capacity [110]. Another beneficial role of BM-MSC-derived exosomes in attenuation of lung injury was attributed to miRNA-425 delivery into lung cells [111].

Furthermore, intraperitoneal administration of human umbilical cord (hUC) and rat BM MSC-derived EVs in mouse models of BPD resulted in diminished lung inflammation, alveolar injury and alveolar-capillary leak, preserved alveolarization and angiogenesis, as well as improved fibrosis and attenuated pulmonary hypertension. A neuroprotective benefit was also evidenced [112,113,114]. The effect was partially attributed to EV enrichment in Tumor Necrosis Factor-Stimulated Gene-6 (TSG-6), a key modulator of immune response, as TSG-6 shifts macrophages from a proinflammatory M1 phenotype to an anti-inflammatory M2 phenotype [112]. Moreover, intratracheal administration of hUC, WJ or BM-MSC-derived EVs in rat BPD models has shown an attenuation of hyperoxic lung injury, as well as enhanced alveolarization and alleviated lung inflammation in a lipopolysaccharide-induced rat BPD model. The therapeutic benefits were mainly attributed to VEGF transfer, increased pro-angiogenic signaling, and anti-inflammatory and anti-apoptotic pathways, while they may extend into long-term effects [115,116,117,118,119,120]. Similar beneficial effects have been demonstrated in a rodent BPD model, utilizing amniotic fluid-derived EVs [121]. An antioxidant function of MSC exosomes has also been clarified as a strong protective mechanism in BPD development [122,160]. Additionally, in vitro and in vivo studies demonstrated that the administration of hUC-derived MSC-EVs prevented the apoptotic death of alveolar type II cells (AT-II), a significant underlying pathophysiologic mechanism in BPD evolution [123,124,161]. Another study by Zhu et al. evaluated the therapeutic potential of amniotic epithelial cell-derived EVs in experimental BPD and demonstrated that EVs from full-term neonates improved lung injury, reduced airway hyper-responsiveness and increased AT-IIs, while they ameliorated pulmonary hypertension and right ventricular hypertrophy. On the other hand, EVs from preterm neonates demonstrated a diminished therapeutic efficacy [105].

Intranasal administration of hUC-MSC exosomes, as well as BM-MSCs-EVs and BM-MSCs-EVs-miR-34c-5p treatment in mouse BPD models, have also yielded favorable results, considering lung mechanics, such as reduced resistance and improved compliance [125,162]. A preventive modality of EVs in BPD has also been identified, since antenatal treatment with human BM-MSCs-EVs suppressed inflammatory cytokines and preserved lung growth and mechanics in a rat model of chorioamnionitis-induced BPD [126]. The preventive and therapeutic utility of exosomes has been unveiled regarding human breast milk-derived exosomes, which have been shown to be efficacious in downregulating cell apoptosis and repairing hyperoxia-induced AT II cell damage, through inhibition of the IL-17 signaling pathway and promotion of DNAJB6 gene transcription [127,163].

Besides BPD, EVs may serve as novel therapeutics even antenatally in lung hypoplasia, as the administration of amniotic fluid stem cell-derived EVs in rat fetal models of pulmonary hypoplasia has been effective in rescuing lung development, regenerating lung epithelium and mesenchyme and promoting branch morphogenesis [164,165].

In conclusion, EVs have strong potential prospects in BPD management. Notably, the benefit of EVs may not be limited to the direct neonatal period, but may expand into childhood and established BPD, as evidenced by experimental studies [128,158]. The therapeutic properties of EVs are attributed mainly to their miRNA cargo, e.g., miR-185-5p, miR-21-5p, as well as their protein content. Proteomic analyses have revealed multiple antioxidant proteins, as well as proteins that control several key pathways, such as inflammation, immune modulation and free radical scavenging. Nonetheless, the bioactive components of EVs probably act synergistically in order to provide the beneficial effect [121,128,129,130].

Despite these promising findings, the predominance of preclinical evidence and the variability in EV source, dose, route, and timing of administration currently limit the direct translation of these findings into neonatal clinical practice.

### 4.2. The Therapeutic Potential of EVs in Neonatal CNS Disorders

Several studies on MSC administration in neonatal CNS disorders like HIE have elucidated that MSCs exert their neuroprotective action mainly through paracrine factors, such as EVs [3]. Multiple studies have investigated the implications of human BM-MSC-EV administration in mice after a hypoxic–ischemic (HI) insult and demonstrated suppressed neuroinflammation and substantial neuroprotection, as evidenced by reduced microglial activation and astrogliosis, diminished endothelial activation, cell death and tissue loss, as well as enhanced neurotrophic growth factor expression and oligodendrocyte maturation [131,132,133,134]. A beneficial effect of human BM-MSC EVs was also identified on brain stem function and hypomyelination in a fetal ovine HIE model [135]. The neuroprotective properties of MSC-EVs (placental or human BM-derived) are attributed to phosphatidylinositol 3-Kinase/AKT signaling and prevention of cytosolic calcium overload, preservation of BBB integrity through the Annexin A1/Formyl peptide receptor (ANXA1/FPR) pathway, as well as through modulation of the mitogen-activated protein kinase (MAPK) and NF-kB signaling pathway and consequent suppression of inflammation [135,136,137,166]. Downregulation of the pro-inflammatory cytokine tumor necrosis factor a (TNFa) and upregulation of the anti-inflammatory cytokine TGF-β by human BM-MSC EVs has also been evidenced, while an additional protective mechanism of hUC-derived MSC-EVs is mediated through FOXO3a expression, increased mitophagy and pyroptosis inhibition [134,138]. Furthermore, miR-124-3p and miR-17-5p encapsulated in astrocyte-derived EVs ameliorated HIE-induced brain injury in neonatal mice through modulation of immune response, anti-apoptotic and antioxidant effects [139,167]. Moreover, miR-21a-5p in BM-MSCs-EVs exerted a neuroprotective role in HIE-induced injury in neonatal mice, while miR-let-7-5p in human WJ-MSCs-EVs demonstrated an anti-apoptotic capacity through caspase 3 regulation [140,141]. Similarly, caspase 2 was targeted by miR-150-3p in neural stem cell exosomes, leading to suppression of neuronal apoptosis [142]. An anti-apoptotic effect, along with increased neuronal viability, was produced by BM-MSC EVs miR-410 in neonatal mice with HIE-induced brain damage [168]. Finally, miR-93 packaged in BM-MSC EVs ameliorated brain injury in neonatal mice with HIE through the JMJD3-dependent p53/KLF2 axis, while miR-7b-5p of hydrogen sulfide-preconditioned MSC EVs promoted an anti-inflammatory microglia and mononuclear phagocyte phenotype [143,144]. In conclusion, EVs may serve as an adjunctive modality to therapeutic hypothermia in neonates with HIE and augment not only short-term, but also long-term neurodevelopmental outcomes.

With regard to periventricular leukomalacia (PVL), a preterm-related white matter injury (WMI), EVs may act as a crucial protective and therapeutic mechanism. Administration of human WJ-MSCs-EVs and BM-MSCs-EVs in a rat model of perinatal brain injury resembling PVL resulted in restored myelination, rescued mature oligodendroglial and neuronal cell counts, suppressed microgliosis and diminished reactive astrogliosis, as well as improved functional recovery [145,146]. Moreover, human WJ-MSC exosomes led to less microglia-mediated neuroinflammation in rats with perinatal brain injury, while in an in vitro model of neuroinflammation, exosomes diminished the expression of inflammation-related genes and secretion of pro-inflammatory cytokines through interference within the Toll-like receptor 4 (TLR4) signaling pathway [147].

The implication of hUC-MSCs-EVs in IVH has also been explored and mitigation of IVH-induced neuroinflammation and cell apoptosis, as well as prevention of post-hemorrhagic hydrocephalus, has been demonstrated, partially through brain-derived neurotrophic factor (BDNF) [148]. Finally, the therapeutic efficacy of BM-MSCs-EVs was investigated in a mouse model of perinatal arterial ischemic stroke. MSC-EVs were accumulated by microglia in the injured cortex and downregulated injury-induced inflammatory cytokines and chemokines [149].

Collectively, these findings support the neuroprotective potential of EVs across different forms of neonatal brain injury, although evidence remains predominantly preclinical and their long-term neurodevelopmental benefit has yet to be established.

### 4.3. The Therapeutic Implication of EVs in NEC

As aforementioned, NEC represents a life-threatening condition, with potentially devastating long-term consequences. Stem cell and EVs-based therapies are a substantial target in NEC, since they provide epithelial regeneration, barrier integrity, anti-inflammation and immunomodulation, as well as angiogenesis [169]. The preventive utility of EVs has been demonstrated in rat NEC models, as MSC-EVs from various sources (such as BM and amniotic fluid) reduced NEC incidence and improved intestinal permeability and wound healing [150,151]. Moreover, Li et al. identified a stimulation of intestinal recovery by amniotic fluid stem cells and their EVs in a mouse NEC model, via activation of the Wnt signaling pathway, an integral pathway in tissue homeostasis and regeneration. Interestingly, the timing of therapy was crucial, as the rescuing effect was demonstrated only when EVs were given during NEC induction and not prior to it [152].

Nevertheless, the utmost benefit of EVs on NEC originates from human milk-derived EVs (HMEVs). Human milk represents a plentiful source of EVs, particularly colostrum [170]. HMEVs exert multiple protective mechanisms, such as modulation of inflammation, maintenance of intestinal barrier integrity and attenuation of epithelial injury. The anti-inflammatory properties of HMEVs consist of IL-6β and TNF-α reduction, two cytokines with significant impact on NEC development due to activation of the TLR4 pathway. Furthermore, HMEVs inhibit activation of TLR3 and TLR9, while they suppress T cell activation. The protective impact on the intestinal barrier is partially ascribed to promotion of cell migration through the p38 MAPK pathway, as well as an increase in epithelial tight-junction proteins [171,172,173]. Moreover, HMEVs promote cell growth and proliferation, particularly exosomes from preterm human milk, attenuate intestinal epithelial cell death and confer antioxidant protection in NEC, probably via the Wnt/b-catenin signaling pathway [174,175,176,177,178]. Additionally, HMEVs alleviated NEC in a mouse model of disease through restoration of ileal crypts and inhibition of the inflammatory process, while amniotic fluid stem cell-derived exosomes exerted their protective role via regulation of epithelial cell migration. A beneficial effect on NEC-associated brain injury was also identified [153].

The advantageous role of HMEVs arises from their protein cargo, such as lactoferrin, omega-3 fatty acids, mRNA, long non-coding RNA (lncRNA) and miRNA content [177,179,180]. MicroRNA (miRNA) cargo of HMEVs is shared between mammalian species, indicating an evolutionary protective procedure [181]. Evidence indicates that miRNA abundance in human milk is stable upon digestion, while a large proportion of miRNA is related to synapse development and immune response [179]. Moreover, miR-148a-3p in human breast milk exosomes exerts anti-inflammatory and anti-apoptotic action in a NEC mouse model, through the miR-148a-3p/p53/SIRT1 axis [182]. Another advantage of human milk exosomes is that they are preserved following the freeze–thaw cycle, while theoretically they could be administered to the NEC patient even when nil per os is applied [183]. Unfortunately, pasteurization leads to a 50% decrease in exosome concentration due to their degradation [184]. Nevertheless, commercialized production of human milk-derived exosomes could be an alternative in cases of inadequate human milk supply.

Overall, these findings support the therapeutic potential of EVs in NEC, while the apparent dependence of their efficacy on the source, timing of administration and processing of human milk highlights important considerations for their future clinical translation.

### 4.4. The Therapeutic Potential of EVs in ROP

Retinopathy of prematurity (ROP) is a potentially blinding disease, currently managed with laser photocoagulation, VEGF inhibitors, or even scleral buckling and/or vitrectomy in exceptional cases. However, the risk of vision problems is not abolished, while less invasive methods are required and EV-based therapies may fill this gap [185]. Intravitreal injection of microglia-derived exosomes in a mouse model of oxygen-induced retinopathy demonstrated a positive effect on neovascularization and improved oxygen-induced retinal vaso-obliteration, in association with suppressed VEGF expression. Visual injury and photoreceptor apoptosis were also ameliorated. The beneficial effect was mainly ascribed to exosomal miR-24-3p [154]. Human BM-MSC-derived exosomes were intravitreally administered in a similar mouse model of oxygen-induced retinopathy and resulted in a lesser degree of retinal ischemia and neovascularization, preservation of retinal blood flow and reduction in retinal thinning [155]. Additionally, administration of MSC-EVs into vitreous humor following retinal ischemia in a rodent disease model led to functional recovery through anti-apoptotic and anti-inflammatory pathways. Moreover, human BM-MSC-derived EVs remained in the vitreous humor for as long as 4 weeks after the injection, indicating a long-lasting effect [156]. Finally, a combined in vitro and in vivo animal study demonstrated a strong anti-angiogenic effect of lymphocyte-derived microparticles, partially due to the miR-181a cargo [186].

Although these findings suggest that EVs may modulate retinal angiogenesis and injury, the available evidence is exclusively preclinical, and the potential consequences of manipulating angiogenic pathways in the developing retina require careful evaluation before clinical translation.

## 5. Comparability and Limitations of the Available Studies

Despite the promising and optimistic results of the available studies, certain limitations exist. First of all, the heterogeneity between studies is not negligible and there is an urgent need for the establishment of standardized and straightforward protocols. Although most studies have used MSC-derived EVs due to the anti-inflammatory, antioxidant, regenerative and immunomodulatory properties of MSCs, there are substantial differences among studies. Extracellular vesicles (EVs) have been derived from MSCs from different donors and tissues and at distinct ages, hampering the comparability of the results. For instance, umbilical cord blood-derived MSC-EVs obtained from preterm donors at 25 weeks of gestation were more effective in reducing neonatal lung injury, compared with donors at 30 weeks of gestation [112]. Cell culture conditions are also important and specific factors influence the profile of the released vesicles, such as the culture system, media composition and oxygen tension [187]. Additionally, some investigators have used bioreactors to expand MSCs, while others utilized conventional dishes or flasks.

The available studies have applied different isolation techniques of EVs, which may directly influence the EVs’ bioactive potential. Although ultracentrifugation is the most widely used isolation method, different spinning speeds have been used, limiting the generalizability of the results. Many studies did not fulfill the characterization criteria of EVs proposed by MISEV guidelines. Future studies should adhere strictly to these recommendations in order to identify the most effective EV population for each disease entity. The use of fresh or stored EVs was not mentioned in many studies, as well as whether the EVs were characterized before use or before storage. The concentration and stability of EVs are influenced by storage conditions, particularly temperature and buffers. Additionally, the disease models varied significantly among studies. Although most studies on HIE applied the Rice–Vannucci model, studies on BPD used varied oxygen concentrations and exposure times, while others used endotoxin exposure. Furthermore, great heterogeneity exists with regard to dosage, route and time of administration, while the organs and tissues of interest were assessed at different timepoints and with variable outcome measures.

Conclusively, the high variability among studies with regard to disease model, EVs source, isolation techniques, dosage, route and time of administration, as well as timepoints for outcome measurement, hinders the generalizability and reliability of the studies’ results.

Similar challenges exist with regard to the studies of EVs as diagnostic biomarkers in neonatal diseases. The significant diversity among the available neonatal studies regarding gestational and chronological age, the biological fluid, the sampling time, as well as the processing methods, limits the comparability of the results. Additionally, a lot of previous studies lack adherence to MISEV requirements, representing a substantial limitation. Moreover, the absence of specific neonatal reference ranges and standardized detailed platforms represents a substantial obstacle for the clinical application of EVs as diagnostic or prognostic biomarkers.

## 6. Future Perspectives and Implications for Research

The awareness of EVs’ implications in neonatal diseases, both as biomarkers and therapeutic tools, is expanding rapidly, but several issues hamper their transition from bench to bedside.

One of the main challenges is the production of homogeneous MSC-EV preparations, based on straightforward criteria for MSC-EV isolation, purification and characterization, as proposed by MISEV guidelines [8]. Another important step is the assessment of the MSC-EVs’ potency and purity prior to their clinical use, based on a reliable functionality method. Particular differences have been observed between EV preparations, regarding the particle number, potency and purity that may affect the functionality [188]. Moreover, the EV source is essential, since large-scale production of effective EVs is mandatory for clinical application. As aforementioned, EVs represent a fingerprint of the cell of origin; thus, the identification of the best cell source is integral. A master bank should be developed in order to establish an adequate number of well-characterized cell sources [106]. Importantly, EVs derived from different cellular or tissue sources cannot necessarily be considered biologically equivalent, as their molecular cargo and biological activity may be influenced by their cell of origin, donor characteristics, and particularly gestational age [189,190]. Cell characterization is required to evaluate cellular properties like purity, viability and functionality and can be validated by flow cytometry, gene expression analysis and functional potency assays. Additionally, the cell culture environment should be optimized with careful monitoring of the expansion and cultivation of cells under controlled laboratory conditions, as well as monitoring of cell behavior, since they may affect EV composition and yield [106]. Three-dimensional (3D) culture systems (bioreactors) can generate greater EV yield compared with two-dimensional (2D) systems (dishes or flasks). The discrepancy may be up to 40-fold in EVs per mL of conditioned media [191]. Cell density is also important, as low densities are associated with increased EVs production. Thus, the use of cell equivalents to quantify EVs may be inappropriate, as cell density better determines the EVs yield, rather than absolute cell numbers. Moreover, EVs bioactivity is reduced with increased cell passage, especially beyond passage 4. Cell aging results in progressive shortening of chromosomal telomeres and a subsequent shift in EVs from a protective profile (low cell passage) to a pro-inflammatory state (high passage). Media content also determines the EV profile, while priming cells with soluble growth factors may influence EV bioactivity [189]. Multiple techniques may enhance the therapeutic efficacy of EVs, for example, preconditioning cells to generate EVs with optimized properties, such as anti-inflammatory, pro-angiogenic and antioxidant potential. Genetic modification and reprogramming of the cell source may lead to the bioengineering of EVs with specific bioactive efficacy or even vehicles for gene therapy [189]. Additionally, bioengineering strategies include direct EV surface functionalization, particularly the addition of targeting moieties and fluorescent dyes to enhance their specificity, internalization and traceability. Functionalization of EVs surfaces further utilizes transporting extramembrane molecules for therapeutic purposes; however, there are concerns about instability of these cargos and, generally, luminal loading is preferred [192]. A master cell bank can be created either by pooling of allogeneic donors or use of genetically engineered cell lines. The first option is fast and cost-effective; however, there are several hurdles, like a contamination risk, variability, lack of clonal stability and heterogeneity. The second option is to create an immortalized master cell clone, which offers homogeneity, controlled variability, reduced contamination risk and allows for targeted approach through the manipulation of therapeutic molecules. Significant drawbacks of utilizing genetically modified cell lines are the necessity for increased regulatory oversight and safety concerns, such as off-target effects, tumorigenesis and immunogenicity [106]. Additionally, the EVs characterization should be standardized according to MISEV guidelines [188].

Multiple isolation techniques have been used, such as density gradient centrifugation (DGC) and differential or sequential ultracentrifugation (dUC), low-to-high speed. The ideal technique would provide a purified EV population with high yield and specificity, but this goal remains currently unrealistic. The available methods are significantly heterogeneous regarding EV concentration, surface markers, proteomic profile and particle size distribution. Thus, the isolation technique can remove key therapeutic components and directly affect the bioactive potential. The implementation of a standardized isolation protocol, probably combining multiple techniques, is imperative for the development of homogeneous, specific and reproducible EV products that can be clinically applied [189,190]. Currently, there is no gold-standard isolation technique. Ultracentrifugation is a commonly applied method, but it is lengthy and depends on inter-operator variability. Alternatively, the use of size-based techniques, like size exclusion chromatography, is increasing. Moreover, ultracentrifugation is disadvantageous in maintaining batch-to-batch consistency, a key factor for the clinical translation of EVs [193]. There is no one-size-fits-all method, but the ideal technique should be fast, automated, simple and able to yield high-quality EVs reproducibility [106].

Another concern is the production of EVs, which necessitates the implementation of large-scale production strategies and industrialized manufacturing. Interventions to enhance production or modify EV content to influence their functional and therapeutic potential are of great interest and include 3D cultures, chemical stimulation, genetic transformation, physical stimulation (like ionizing and non-ionizing radiation) and physiological manipulation (like hypoxia, oxidative stress and heat shock). These methods are so far superior to 2D cultures and should be carefully implemented. Another matter of consideration is the determination of EV biodistribution in vivo. Several methods have been implicated, each with its inherent limitations. Fluorescently labeled EVs are the most widely used method, mainly due to their simplicity. However, particular disadvantages are present, like the potential leaching of fluorescent dyes that may lead to background signals, non-specific dye transfer between membranes and low spatial and temporal resolution. Radiolabeled EVs represent an alternative method, with high labeling efficiency, high sensitivity and depth penetration. On the other hand, there is radiation exposure, the cost is high, and analysis at long timepoints is limited, while the EVs’ surface properties may be affected. Another available method is bioluminescence labeling, which is an efficient and sensitive method; however, it requires cell engineering, lacks deep tissue penetration and can modify EVs’ protein composition. Notably, each method may affect EVs’ surface proteins and/or composition with a direct impact on their therapeutic efficacy, particularly exogenous EVs modification [106].

Clinical application of EVs would necessitate “ready-to-use” products; thus, the preservation and storage conditions should be optimized in order to maintain the EVs’ bioactivity. The most widespread preservation method for viable cells and tissues is cryopreservation. Extracellular vesicles (EVs) are usually preserved at −20 °C to −80 °C in phosphate-buffered saline. However, further research is required regarding base buffers and cryoprotectants, especially due to the known infusion toxicity of certain cryoprotectants. Additional methods of preservation are lyophilization, freeze-drying, and spray drying, which could offer the advantage of long-term storage at ambient temperature and increase the shelf life and scalability [106]. The establishment of reproducible quantification strategies is mandatory to measure the therapeutic effects of EVs. Most studies rely on measurement of total protein amount, but the effective doses vary considerably. For instance, a range from 0.001 to 100 mg of EV protein per kilogram of body weight has been demonstrated. The alignment of EV doses with qualitative assessment of EVs may be the most appropriate strategy [106,194]. Despite the ongoing research on EVs, the large-scale industrial manufacturing of them is still in its infancy and many aspects should be addressed.

Understanding the pharmacokinetics of EVs, especially in the exceptional neonatal population, is of paramount importance in order to clarify the dosage and frequency of administration. The optimal dose, the appropriate route of administration, timing, and biodistribution are essential, since they exert an impact both on the efficacy and the safety of the applied modality [189,190]. Notably, preclinical studies have shown a beneficial effect of an early single dose of MSC-EVs, but other studies have shown efficacy with multiple doses during the later stages of the disease process [188]. For example, intravenous injection of EVs in animal models led to localization of large EVs in lung tissue within one hour, while the small EVs were detected after 2–12 h [159]. It is yet unclear whether this time period is adequate for EVs to transfer their cargo to the recipient cell and for how long EVs remain in the lungs. Thus, it is uncertain if a single dose is enough to provide short- and long-term efficacy or if multiple doses are required. Moreover, the application of a weight-based dosage formula is probably the best approach in the neonatal population. The selection criteria for patients to be treated are another issue of consideration. Treating the most severely ill babies in the setting of the complications that may develop, like scarring and fibrosis in BPD, may not be rational. Early treatment of patients who will not finally progress to a severe disease state may be unnecessary [188].

Despite the high potential of EVs, some concerns have arisen about the possible toxicity of nanoparticles in vivo and ex vivo. Further research is also warranted in order to investigate the potential relation of EVs to thrombotic complications, as exosomes carry TF and PS on their surface, well-known activators of the coagulation process. Pre-incubation of EVs with heparin may counterbalance their prothrombotic properties. The development and validation of in vitro potency tests is mandatory to check EV preparations before clinical application and ensure a safety profile. The combination of various therapeutic modalities should also be investigated, as a mouse model of HIE demonstrated diminished efficacy of cell therapy in cases pretreated with hypothermia [3,189,195].

A deeper deciphering of the mechanism of action of EVs is also required, while the sorting of protein and lipid cargo should be further elucidated. Advanced analytical methods like proteomic and genomic profiling are highly required to fully reveal the identity and the quantity of the EVs’ active therapeutic cargo. Indeed, the exact composition of EVs and the associated mechanism of action are not fully elucidated. For example, the role of non-coding RNAs (e.g., piRNA and circular RNAs) and double-stranded DNA necessitates further ascertainment [3]. Despite the extensive research in the field, the effector component of EVs has not been fully elucidated. Most studies indicate that miRNAs or single proteins are the active molecules of EVs, but probably the beneficial effect is attributed to an orchestrated combination of their diverse and rich cargo, rather than a single moiety. With regard to perinatal pathophysiology, the greatest advantage of EVs lies in immunomodulation. Extracellular vesicles (EVs) modulate immune cell activation, leading to vascular stability, parenchymal/epithelial support and, finally, to tissue repair and homeostasis [188]. Another crucial question to be answered concerns the molecular signature of therapeutic EVs, due to the feasibility of EV engineering [107]. A significant obstacle is the lack of standardized criteria for EV production [196]. Further standardization is also required regarding EV purification, storage and stability, dose normalization, and potential batch-to-batch variability. The development of validated potency assays will be essential to ensure the consistency and biological activity of therapeutic EV preparations.

The main clinical studies regarding the therapeutic efficacy of EVs were conducted in COVID-related Acute Respiratory Distress Syndrome (ARDS) and, though animal models are useful, the research must focus on large-scale clinical, double-blinded, placebo-controlled studies. This is imperative, as particular differences take place between mouse and preterm models, such as simpler lung architecture and distinct postnatal alveolarization in mice [125]. With regard to EVs-based BPD studies, term rodents are usually used, which are born at the mid- to late-saccular stage of lung development, in alignment with a gestational age of 30 to 36 weeks in humans. Different animal models are required that are equivalent to earlier human gestation, corresponding to the saccular or even the canalicular stage of lung development. Indeed, nowadays, severe BPD affects mostly neonates born at the canalicular stage of lung development [189]. Moreover, some biomarkers may indicate usefulness in preclinical studies, but fail to be valid in clinical trials [185]. To the best of our knowledge, certain clinical trials about EVs in neonatology have been registered, exploring the role of EVs in BPD, prematurity-related brain injury, HIE and NEC prevention, but no results have yet been yielded [3,196]. A summary of the clinical trials of EVs in neonatal diseases is presented in Table 8.

Furthermore, EVs may be the next-generation drug delivery system, as they may be used as “drug vehicles” [197]. Extracellular vesicles (EVs) may target a particular tissue or organ, while avoiding off-target actions [198]. The “drug cargo” can be loaded into the EVs and the EVs can be engineered to augment their targeting and biomimetic properties. Moreover, EVs exhibit low immunogenicity and do not replicate after injection; thus, the transfer of latent viral pathogens and tumorigenesis are less likely. Additionally, EVs are superior to synthetic drug carriers in penetrating tissues and cell barriers, while they are less likely to induce adverse events due to their origin from biological or autologous origin [199]. On the other hand, significant obstacles exist. For example, one study demonstrated rapid EV distribution and retention in organs like the lungs and liver after intravenous injection, while another study showed rapid clearance of EVs with a half-life of 7 min [200,201]. Additionally, EVs have been proposed as gene delivery platforms for gene therapeutics, although they have major limitations and pose significant translational challenges [202].

The next step in EV engineering may be the development of artificial EVs, currently under investigation. Two approaches are employed, namely the top-down and the bottom-up approaches. The first one relies on the production of membrane fragments from the disruption of cultured cells. These fragments will form vehicles while preserving the membrane features of the initial cell. The second approach is based on the creation of complex structures starting from small molecular blocks, leading to synthetic EVs [203,204].

Moreover, EVs-based diagnostics cannot be generalized, as EVs release and cargo hypothetically vary between patients and are influenced by many factors, like genetics, age and sex. The development of personalized EVs diagnostics may be more appropriate [193].

The cost of these diagnostic and therapeutic modalities should also be considered, while the safety and efficacy should be safeguarded. The long-term safety of EVs therapeutic application in the developing organism is of paramount importance, as the potential impact on developmental, immune and angiogenetic pathways may not be evident during the short-term follow-up.

The rationale for a regulatory framework is of high priority, where researchers could submit details and possible deficits of their research process and a community network could facilitate the current state-of-the-art on EV studies.

## 7. Conclusions

The field of EVs represents a rapidly evolving area of neonatal research, with emerging evidence supporting their potential roles as biomarkers, mediators of disease pathophysiology, and therapeutic tools. Preclinical studies demonstrating protective effects across multiple organs, including the developing lung, brain, intestine, and retina, highlight the broad therapeutic potential of EV-based approaches. Nevertheless, substantial challenges remain before their translation into neonatal clinical practice, including the identification of the optimal EV source, dose and route of administration, standardization of isolation and characterization methods, and, importantly, demonstration of long-term safety and efficacy.

Among naturally occurring sources of EVs, human milk is of particular interest because of its rich EV content and the emerging evidence supporting their contribution to intestinal, immune, and developmental protection, further reinforcing the importance of breastfeeding and human milk provision in the NICU. In parallel, the distinct qualitative and functional characteristics of neonatal compared with adult-derived EVs further emphasize the developmental specificity of EV biology and raise intriguing questions regarding their potential contribution to developmental mismatch following transfusion of adult-derived blood components.

Thus, in response to the question posed in the title, EVs cannot yet be considered the “magic bullet” of neonatology. However, their unique capacity to reflect disease processes, mediate intercellular communication, and potentially modify injury across multiple developing organs makes them a particularly promising field for translational research. Rigorous preclinical and clinical investigation, together with standardized methodologies and careful assessment of safety, will be essential to determine whether this promise can ultimately be translated into effective and individualized neonatal therapies.

## Figures and Tables

**Table 1:** Extracellular vesicle profiles in relation to gestational age.

Authors, Year, Disease Entity	Study Population	Gestational Age (Weeks)	Sampling Timepoint	Biofluid	EVs Origin EVs Subtype and Size	Key Findings
Murphy, 2025 [35] Prematurity	Total: 167 N: 101 preterm N: 66 term	Preterm: 28.3 (26.4–29.9) weeks Term: 39.29 (38.9–39.9) weeks	Preterm group: At birth (UCB) Day 1 (0–24 h of life) Day 3 (48 72 h of life) Day 14–21 Term group: At birth (UCB) Day 1: 0–24 h of life Day 3: 25–96 h of life	Plasma (UCB and peripheral blood)	Platelets Endothelial and leukocyte cells SEVs (<200 nm) LEVs (1000–1300 nm)	EV markers: CD63, CD81 PS exposure: Annexin V Platelet origin: CD41 (GPIIb), CD42b (GPIb), CD62P (P-selectin) Endothelial origin: VEGFR2 Leukocyte origin: CD45 Procoagulant marker: TF Preterm group: Augmented expression of platelet-, endothelial- and TF-positive EVs during the first 3 days of life and a distinct proteomic profile, associated with coagulation activation, pulmonary pathophysiology and immune response.
Ohta, 2022 [36] Prematurity	Total: 20 preterm infants	34.1 (±1.4) weeks	At birth and at 2, 4, 8, 24, and 48 weeks of age	Serum (peripheral blood)	EV origin not determined Exosomes	EV markers not determined Exosomes increased steadily during the 1st year of life. Exosome levels correlated positively with gestational age and multiple birth, while respiratory disease had no impact.
Bruschi, 2018 [37] Prematurity	Total: 6 N: 3 Term N: 3 Preterm	Term: >37 weeks, Preterm: 28–32 weeks	At birth	HUCMSC culture supernatant	HUCMSC Microvesicles	EV markers (identity markers): Mitochondrial-associated proteins: ATP5B, COX5A Proteomic analysis: 173 differentially expressed proteins between preterm vs. term. Preterm MVs enriched in: Oxidative phosphorylation proteins, TCA cycle enzymes, Electron transport chain subunits (Complex I–V), Functional assays: Both term and preterm microvesicles consume oxygen, but only preterm microvesicles synthesize ATP. Distinct biological processes associated with oxidative metabolism and inflammatory pathways are upregulated in the preterm group.
Dixon, 2018 [38] Prematurity	Total: 45 mother/fetus pairs N: 13 PTB N: 8 PPROM N: 11 TL N: 13 TNIL	Term: 37–42 weeks, Preterm: <37 weeks	During labor or prior to cesarean section	Amniotic fluid	Placental, intrauterine tissues Exosomes	EVs identity: CD63, PLAP Significantly higher levels of PLAP (+) exosomes in PTB, PPROM, TL group compared to TNIL Proteomic analysis: Differential expression of proteins involved in inflammation, immune response and oxidative metabolism. Consistent expression of proteins involved in cellular movement, cell-to-cell signaling, cell death and survival. AF exosomes reflect the intrauterine environment at time of birth, under particular circumstances and verify known biological processes during labor, such as inflammation irrespective of gestational age, fetal membrane cell aging and cell death.
Menon, 2019 [39] Prematurity	Total: 45 mother/fetus pairs N: 13 PTB N: 8 PPROM followed by preterm delivery N: 11 TL N: 13 TNIL	Term: 37–42 weeks Preterm: 34–37 weeks	At birth, after labor onset	Maternal plasma	EV origin not determined Exosomes	EVs identity: CD63, TSG-101 and CD9 No difference in total exosome concentration among the groups Proteomic analysis: 72 differentially expressed proteins among the groups, mainly associated with lipid metabolism, molecular transport and inflammation. Non-specific inflammation is common in PTB and term birth, but it is overexpressed in PTB, along with hematological dysfunction. Maternal exosomes reflect deviations in functional pathways that may lead to various pregnancy outcomes.
Menon, 2022 [40] Prematurity	Total: 45 neonates N: 13 PTB N: 10 PPROM followed by preterm delivery N: 10 TL, N: 12 TNIL	Term: 37–42 weeks, Preterm: <37weeks	At birth	Plasma (UCB)	EV origin not determined Exosomes	EVs identity: CD81, CD63, TSG-101 (ESCRT complex), FLOT-1, ICAM1, Annexin A5, Negative marker: GM130 (not contaminated by cellular organelles or other EVs) Proteomic analysis: 786 proteins were differentially expressed. Proteomic profile varied by condition (e.g., upregulated dendritic cell maturation and intrinsic prothrombin activation pathway in TNIL versus TL, downregulated senescence pathway in PTB versus TNIL). An overall inflammatory response is identified; however, specific mechanistic or inflammatory pathways are lacking in UCB exosomes, thus their utility as biomarkers of a (patho)physiologic condition is limited.
Cantonwine 2016 [41] Preterm delivery	Total: 75 mother/fetus pairs N: 25 singleton cases of SPTB ≤ 34 weeks N: 50 term deliveries	Term: ≥37 weeks Preterm: ≤34 weeks	10–12 weeks of gestation	Maternal plasma (peripheral blood)	Multiple potential origins, such as endothelial, invading trophoblast and trophoblastic EVs. Microparticles	Proteomic analysis: Protein biomarkers in SPTB cases linked to coagulation, fibrinolysis, immune modulation and the complement system. Microparticles’ protein contents may serve as early predictors of SPTB ≤ 34 weeks.
McElrath, 2019 [42] Preterm delivery	Total: 261 mother/ fetus pairs N: 87 cases of SPTB ≤35 weeks N: 174 term deliveries	Term: ≥37 weeks Preterm: ≤35 weeks	10–12 weeks of gestation	Maternal plasma (peripheral blood)	EV origin not determined Microparticles	Proteomic analysis: Protein biomarkers in SPTB cases associated with coagulation/wound healing, complement/adaptive immunity, fibrinolysis, lipid metabolism and inflammation/oxidative stress. Microparticles’ protein cargo may serve as a biomarker for risk stratification of SPTB ≤ 35 weeks, especially in nulliparas.
Miranda, 2018 [21] Fetal Growth Restriction	Total: 30 neonates N: 10 IUGR N: 10 SGA N: 10 controls	Controls: 40.1 (39.2–40.6) weeks SGA: 39.4 (38.1–40) weeks IUGR: 38.7 (37.6–40.1) weeks	At birth	Plasma (UCB)	Placental Exosomes	EVs identity: CD63, TSG-101, Flotillin-1, PLAP (placental marker). Placenta-derived exosomes were reduced in SGA and IUGR neonates vs. controls in maternal and fetal plasma, according to disease severity.

Abbreviations: AF: amniotic fluid; ATP, adenosine triphosphate; ATP5B, ATP synthase b-subunit; COX5A, cytochrome c oxidase subunit 5A; EVs, extracellular vesicles; GM130, Golgi matrix protein 130; HUCMSC, mesenchymal stem cells from human umbilical cord; ICAM1, intercellular adhesion molecule 1; IUGR, Intrauterine Growth Restriction; LEVS, Large Extracellular Vesicles; MVs, microvesicles; PLAP, placental alkaline phosphatase; pPROM, preterm premature rupture of membranes; PS, phosphatidylserine; PTB, preterm birth; SEVs: small extracellular vesicles; SGA, Small for Gestational Age; SPTB, Spontaneous Preterm Birth; TCA, Tricarboxylic Acid Cycle; TF: tissue factor; TL, term labor; TNIL, term not in labor; TSC-101, tumor susceptibility gene 101; UCB, umbilical cord blood; VEGFR2, Vascular Endothelial Growth Factor Receptor 2.

**Table 2:** The role of EVs as diagnostic biomarkers of the neonatal hemostatic mechanism.

Authors, Year Disease Entity	Study Population	Gestational Age (Weeks)	Sampling Timepoint	Biofluid	EVs Origin EVs Subtype and Size	Key Findings
Michelson, 2000 [52] Hemostasis	Total: 28 N: 7 Term neonates N: 8 Preterm neonates N: 13 adults	Term: 38–41 weeks Preterm: 24–30 weeks	Neonates: at birth Adults: single timepoint	Plasma (UCB & peripheral blood)	Platelets Microparticles	EVs identity: GPIb (CD42b) Procoagulant activity was determined by binding of Factor V/Va antibody. Increased levels of PEDVs were demonstrated both in term and preterm neonates in comparison to adults. The procoagulant activity of PEDVs after activation was diminished in preterm neonates compared with term newborns and adults, due to relatively diminished Factor V (FV) levels.
Schmugge, 2003 [53] Hemostasis	Total: 94 N: 38 neonates (UCB) N: 19 neonates (peripheral blood samples) DOL: 2–3 (15 of which were repeat samples from the UCB group, 4 were studied only once) N: 15 Children N: 22 Adults	Neonates: 36–42 weeks	Neonate: At birth DOL: 2–3 Children Adults: Single timepoint	Plasma (UCB & peripheral blood)	Platelets Microparticles	EVs identity: CD41 (GPIIb) PS exposure: AnnexinV Neonatal platelets demonstrated increased baseline activation and higher levels of platelet microparticles in neonates (particularly on DOL 2–3) compared with adults. Platelet-derived microparticles may serve as biomarkers of platelet activation and procoagulant activity.
Wasiluk, 2008 [54] Hemostasis	Total: 106 N: 51 preterm neonates N: 55 term neonates	Preterm: 25–36 weeks Term: 38–41 weeks	At birth	Plasma (UCB)	Platelets Microparticles	EVs identity: CD61 (GPIIIa/GPIIb-IIIa complex) Higher levels of platelet-derived microparticles in the preterm group (especially in males) compared with the term group, probably serving as a compensatory hemostatic mechanism.
O’Reilly, 2018 [55] Hemostasis	Total: 21 Preterm neonates (8 neonates with matched samples on DOL 1 and 3)	Not determined	DOL 1 and/or 3	Plasma (peripheral blood)	Platelets SEVs, LEVs	EVs identity: CD41+ (GPIIb) Annexin V+: PS exposure Increased levels of SEVs and LEVs from DOL 1 to DOL 3. The levels of platelet-derived EVs decreased from DOL 1 to DOL 3, indicating early platelet activation during the extrauterine transition period.
Schweintzger, 2010 [56] Hemostasis	Total: 59 N: 31 Term neonates N: 28 adults	Term neonates, Gestational age not recorded	At birth	Plasma (UCB & peripheral blood)	Platelets Microparticles	Annexin V+: PS exposure Slightly higher levels of microparticles and a significantly enhanced procoagulant activity were identified in neonates compared with adults, suggesting an implication of neonatal platelet microparticles in thrombin generation.
Schweintzger, 2011 [57] Hemostasis	Total: 59 N: 31 Term neonates N: 28 adults	Term: 38–40 weeks	Neonates: at birth Adults: single timepoint	Plasma (UCB)	EV origin not determined Microparticles	Annexin V+: PS exposure Microparticles were measured at mildly elevated concentrations, but demonstrated a significantly increased procoagulant activity in neonates compared with adults.
Uszyński, 2011 [58] Hemostasis	Total: 56 N: 28 mother–infant pairs N: 28 non-pregnant females serving as controls	Term: 39.3 (38.0–41.6) weeks	At birth	Plasma (UCB & maternal peripheral blood)	EV origin not determined Microparticles	EVs identity: Annexin V: PS exposure, TF & TFPI Increased levels of microparticles and TF, while decreased levels of TFPI were measured in UCB compared with maternal plasma and controls, suggesting that microparticles act as a compensatory mechanism of the immature fetal coagulation process.
Korbal, 2017 [59] Hemostasis	Total: 48, N: 23 preterm N: 25 term	Preterm: 34 (32–36) weeks Term: 40 (39–41) weeks	At birth	Plasma (UCB)	EV origin not determined Microparticles	EVs identity: TF bearing microparticles Higher levels of TF bearing microparticles were measured in preterm UCB plasma compared with term neonates. A negative correlation was found between TF bearing microparticles and gestational age-birth weight, indicating an increased prothrombotic tendency in preterm neonates.
Karlaftis, 2014 [60] Hemostasis	Total: 120 N: 10 term N: 80 children N: 20 adults	Term: >37 weeks	Neonates: DOL1,3 Children & Adults: Single timepoint	Plasma (peripheral blood)	EV origin not determined Microparticles	EV markers not determined. Neonatal microparticles demonstrated lower procoagulant activity compared with other age groups.
Hujacova, 2020 [62] Hemostasis	Total: 12 N: 5 preterm N: 7 term	Preterm: 31.6 (±2.7) weeks Term: 39 (±0.6) weeks	At birth	Plasma (UCB)	Platelets LEVs	EVs identity: CD36+CD41 CD41+CD62 No significant differences in EV levels were found between the preterm and term groups. However, a decreased fluorescence intensity of CD36 and CD62 EVs was demonstrated in the preterm group, indicating a hyporeactivity of neonatal platelets.
Hujacova, 2021 [61] Hemostasis	Total: 30 N: 20 preterm N: 10 term	Preterm: 31.5 (±2) weeks Term: 38.1 (±1.8) weeks	At birth	Plasma (UCB)	Platelets, Activated platelets, Endothelial cells Microparticles	EVs identity CD41+ CD36+: Platelet-derived microparticles CD41+ CD62P+: Activated platelet microparticles CD31+ CD105+: Endothelial microparticles No significant differences in EV levels were demonstrated between the preterm and term groups, and no correlation of EV counts with gestational age was identified. A mildly decreased fluorescence intensity of CD31+ EVs was demonstrated in the preterm group. EVs may serve as biomarkers of platelet and endothelial status in neonates.
Peñas-Martınez J. 2021 [63] Hemostasis	Total: 26 N: 14 Term N: 12 adults	Term: 38–41 weeks	Neonates: at birth, Adults: single timepoint	Plasma (UCB & peripheral blood)	EV origin not determined Exosomes	EV identity: TSG-101, CD41 (GPIIb), CD63 Proteomic analysis: 131 proteins were differentially expressed. Upregulated proteins were involved in pathways like hemostasis, clot formation, complement cascade, etc. Downregulated proteins were mainly protein S and immunoglobulins. Neonatal exosomes were smaller in size and had a lower amount of protein content. The protein content of EVs correlates well with the concentrations of these proteins in blood and indicates that EVs are probably involved in developmental hemostasis.

Abbreviations: DOL, day of life; EVs, extracellular vesicles; FV, Factor V; LEVS, Large Extracellular Vesicles; PEDVs, platelet-derived EVs; PS, phosphatidylserine; SEVs, small extracellular vesicles; TF, tissue factor; TFPI, Tissue Factor Pathway Inhibitor; TSG-101, tumor susceptibility gene 101; UCB, umbilical cord blood.

**Table 3:** Extracellular vesicles (EVs) as diagnostic biomarkers of neonatal respiratory disorders.

Authors, Year Disease Entity	Study Population	Gestational Age (Weeks)	Sampling Timepoint	Biofluid	EVs Origin EVs Subtype and Size	Key Findings
Starke, 2024 [68] Lung/brain axis	Total: 15 N: 7 neonates on FiO_2_ < 30% N: 7 neonates on FiO_2_ ≥ 30% N: 1 adult (Control)	Low O_2_ group (FiO_2_ < 30%): 27.8 (±1.3) weeks High O_2_ group (FiO_2_ ≥ 30%): 24.3 (±0.5) weeks	Neonates: 1 week of age	Plasma (peripheral blood)	Alveolar macrophages Exosomes	EVs identity: CD9, CD63, CD81, CD11b, CD11c, CD206. ASC EVs derived from alveolar macrophages in the High O_2_ group had increased ASC levels. The adoptive transfer of these EVs in mice resulted in lung inflammation, reduced alveolarization and pulmonary vascular development, as well as brain injury.
Go, 2020 [69] BPD	Total: 73 preterm N: 30 BPD group N: 34 No-BPD group	Overall: <32 weeks No BPD: 28 (±0.3) weeks BPD: 25 (±0.3) weeks	At birth and at DOL 28	Serum (UCB and peripheral blood)	EV origin not determined Exosomes	EVs identity: CD9, CD63 EV miRNAs: 62 miRNAs identified; miRNAs universally expressed in both groups. Key differential cargo: miR-21, miR-17, miR-20a, miR-25, miR 30c, miR-146a, etc. EV miR-21 was upregulated in BPD group on DOL28 compared with birth, while it was downregulated in no-BPD group on DOL28 compared with DOL0. Exosomal miR-21 may serve as a potent diagnostic biomarker in BPD, with good AUC 0.850.
Lal, 2018 [70] BPD	Total: 80 neonates N: 18 ELBW intubated at birth N: 12 ELBW for the validation of miRNAs predicting BPD N: 25 Preterm neonates with established severe BPD at 36 weeks PMA N: 25 Term or near-term neonates intubated but without lung pathology	Severe BPD: 25 (±2) weeks Controls: 37 (±2) weeks	At birth (within the first 6 h of life) At 36 weeks PMA	Tracheal aspirates	Airway epithelial cells Neutrophils Exosomes	EVs identity: MUC4 antibodies for epithelial exosomes. CD66 antibodies for Neutrophil-derived exosomes. Higher concentrations of exosomes were measured in neonates with BPD compared with gestational age-matched full-term controls. A specific miRNA signature was demonstrated at birth in ELBW neonates who later developed BPD, compared with those that did not. A decreased expression of miR-876-3p at birth was found to be a potent biomarker of severe BPD development in ELBW infants.
Ransom, 2023 [71] BPD	Total: 34 preterm	Total: 22^+1^–34^+6^ weeks Late canalicular group: 22^+1^–26^+6^ weeks Saccular group: 27–34^+6^ weeks	Not determined	Tracheal aspirates	Epithelial cells, Immune cells, Progenitor cells, Mesenchymal cells EVs	EVs identity: Tetraspanins: CD9, CD63, CD81 Epithelial markers: CD326 (EPCAM), CD133 (PROM1), CD142 (F3), ROR1, CD24, CD29, CD44SSEA-4 Immune markers: CD3, CD14, CD40, CD41b, CD45, CD56, HLA-ABC, HLA-DRDPDQ Higher levels of CD24+ and CD14+ EVs were found in neonates who went on to develop BPD. EVs in tracheal aspirates may be used as biomarkers of BPD development, as well as mediators of inflammatory pathways involved in BPD pathogenesis.
Amatya, 2026 [75] BPD	Total: 30 preterm N: 15 BPD N: 15 no BPD	BPD group: 25 (22–29) weeks No-BPD group: 29 (24–31) weeks	First 7 days of life	Plasma	EVs origin not determined EVs	EVs identity: CD 63, CD9, CD81 Proteomic analysis: A novel 9-protein profile distinguished neonates who developed BPD from those who did not. Heterogeneous nuclear ribonucleoprotein M (HNRNPM) demonstrated the strongest predictive ability for BPD. A novel 9-protein signature associated with BPD development was identified. The biological functions of these proteins involve mRNA splicing, RNA metabolism, and inflammation.

Abbreviations: ASC, apoptosis-associated speck-like protein containing a caspase recruitment domain; AUC, area under the curve; BPD, bronchopulmonary dysplasia; DOL, day of life; ELBW, Extremely Low Birth Weight; EpCAM, epithelial cell adhesion molecule; EVs, extracellular vesicles; HNRNPM, heterogeneous nuclear ribonucleoprotein M; PMA, Post Menstrual Age; UCB, umbilical cord blood.

**Table 4:** Extracellular vesicles (EVs) as diagnostic biomarkers of CNS disorders, NEC and ROP.

Authors, Year Disease Entity	Study Population	Gestational Age (Weeks)	Sampling Timepoint	Biofluid	EVs Origin EVs Subtype and Size	Key Findings
Spaull, 2019 [81] IVH	Total: 3 preterm with PHH	23–24 weeks	Patient 1: DOL 121 Patient 2: DOL 32 Patient 3: DOL25, DOL29, DOL32, DOL54, DOL 124	CSF	EVs origin not determined Exosomes	EVs identity: CD63+, CD81+ EVs miR cargo: miR-9 miR 17, miR-26a, miR-124, miR-1911 A distinct cargo of brain-related mi-RNAs was identified in CSF exosomes, indicating their potential usefulness as biomarkers in IVH progression.
Goetzl, 2018 [83] HIE	Total: 14 Term neonates with acute HIE undergoing therapeutic hypothermia	Term: 38–41 weeks	At 8, 10, 14 h after the initiation of therapeutic hypothermia	Serum (peripheral blood)	Neuronal Exosomes	EVs identity: Contactin-2/TAG1 (neuronal membrane adhesion protein) EVs protein cargo: Synaptopodin, Synaptophysin, Neuron-Specific Enolase, Cytochrome c oxidase subunit IV The neutral or decreasing slope of synaptopodin was correlated with worse outcomes, indicating that neuronal exosomes and their cargo may serve as a “troponin-like” test in HIE.
Pineles, 2022 [84] HIE	Total: 26 neonates with HIE under therapeutic hypothermia	Term/near-term	At 0–6, 12, 24, 48 and 96 h of life	Serum (peripheral blood)	Neuronal Exosomes	EVs protein cargo: synaptopodin, lipocalin-2 (NGAL), pentraxin. Decreasing levels of synaptopodin and increasing levels of NGAL were associated with worse MRI injury score, indicating that neuronal exosomes may serve as prognostic biomarkers in HIE.
Marell, 2019 [85] Neonatal brain iron status	Total: 79 Term/near-term neonates at risk of iron deficiency	Male: 39.6 (±1.2) weeks Female: 39.4 (±1.2) weeks	At birth	Plasma (UCB)	Neuronal Exosomes	EVs identity: CD81, CNTN2 (neuronal-specific exosomal marker) EVs cargo: CNTN2 (neural adhesion protein), BDNF (brain-derived neurotrophic factor) A positive correlation was demonstrated between exosomal CNTN2 and ferritin levels, while a negative relationship was shown between exosomal BDNF and ferritin levels, indicating a potential utility of exosomal proteins as biomarkers of brain iron status.
Tan, 2020 [86] Acute Bilirubin Encephalopathy	Total: 30 neonates N: 20 with acute bilirubin encephalopathy N: 10 controls	Moderate group: 38.60 (±1.40) weeks Severe group: 38.20 (±1.70) weeks Control: 38.40 (±1.34) weeks	At diagnosis (Patients) Single timepoint (Controls)	CSF	EV origin not determined Exosomes	EVs identity: CD9 EV cargo: 291 Differentially expressed proteins enriched in immune and inflammatory signaling pathways, particularly PI3K-AKT, nF-κB and MAPK signaling pathways. Certain differences in CSF EV protein cargo were demonstrated between the patient and the control group, mostly associated with the immune–inflammatory response.
Galley, 2021 [89] NEC	Total: 42 N: 12 Controls N: 13 surgical NEC N: 9 medical NEC N: 8 sepsis	≤34 weeks	T1: During the first 24 h after enrollment (immediately after diagnosis); T2: 2 to 5 days after enrollment (during disease); T3: 6 to 14 days after enrollment (resolution)	Urine	EVs origin not determined EVs	EVs identity: Alix, CD63 EVs miRNA content: differentially expressed EV miRNA cargo between the study groups, particularly SNEC vs. non-NEC sepsis (including miR-376a-3p, miR-31–5p, and miR-518a-3p), MNEC vs. controls (including miR-604), SNEC vs. controls, as well as MNEC and SNEC vs. controls (including miR-5186, miR-139–3p, miR-5703 and miR-604). No differentiation in EV miRNA cargo between MNEC and SNEC groups. The differentially expressed miRNAs were mostly linked to inflammation-associated molecules. Unfortunately, no early discrimination was observed between the NEC and no-NEC sepsis groups. The alterations were observed 6–14 days after the disease onset.
Kanei, 2025 [90] ROP	Total: 47 N: 33 ROP cases (18 required treatment) N: 14 controls without ROP	Treatment requiring cases: 25^+5^ (±3) weeks Non-treatment requiring cases: 29^+6^ (±3) weeks Controls: 33 (±2) weeks	At 34 weeks PMA	Tears	EV origin not determined EVs	EVs identity: CD9, EpCAM (low, epithelial EV marker), CD147 (low) EVs cargo (miRNAs): 202 miRNA differentially expressed in the ROP group vs. the no-ROP group. Differential expression of miRNAs was also identified in treatment-requiring ROP cases versus non-treatment-requiring ROP cases Among the differentially expressed miRNAs, miR-520a-5p demonstrated strong diagnostic accuracy and diagnostic efficacy in discriminating treatment-requiring ROP cases, with a significant dose–response relationship between miR-520a-5p and ROP severity. However, levels of miR-520a-5p were also negatively correlated with gestational age and its impact was directly related to gestational age. miR-520a-5p is integrated in a network of several angiogenic and inflammatory factors, such as VEGF.

Abbreviations: BDNF, brain-derived neurotrophic factor; CNS, Central Nervous System; CSF, cerebrospinal fluid; CNTN2, Contactin-2; DOL, day of life; EpCAM, epithelial cell adhesion molecule; EVs, extracellular vesicles; HIE, hypoxic–ischemic encephalopathy; IVH, intraventricular hemorrhage; MNEC, medical necrotizing enterocolitis; MRI; Magnetic Resonance Imaging; NEC, necrotizing enterocolitis; PHH, post-hemorrhagic hydrocephalus; PMA, Post Menstrual Age; ROP: retinopathy of prematurity; SNEC: surgical necrotizing enterocolitis; UCB, umbilical cord blood; VEGF, Vascular Endothelial Growth Factor.

**Table 5:** Extracellular vesicles (EVs) as diagnostic biomarkers of endothelial dysfunction in neonates.

Authors, Year Disease Entity	Study Population	Gestational Age (Weeks)	Sampling Timepoint	Biofluid	EVs Origin EVs Subtype and Size	Key Findings
Simoncini, 2017 [93] Endothelial dysfunction	Total: 47 N: 29 preterm N: 18 term	Term: >37 weeks Preterm: 24–35 weeks	At birth	Umbilical cord endothelial colony-forming cells	Endothelial cells Endothelial microparticles	EVs identity: Annexin V+ Increased levels of endothelial microparticles in the preterm group and induction of endothelial senescence by them.
Vítková, 2018 [94] Endothelial dysfunction	Total: 26 N: 13 neonates on ECMO N: 13 controls	ECMO: 38.9 (±0.4) weeks Controls: 39.4 (±0.3) weeks	During the first week of life Controls: During blood sampling for mandatory metabolic screening ECMO group: During ECMO support	Plasma (peripheral blood)	Endothelial and mucosal cells Microvesicles	EVs identity: Annexin V Endothelial origin of EVs: CD105, PECAM1/CD31, VEGFR2/(CD309), MadCAM Increased levels of total and endothelium-specific microvesicles in the patient group vs. healthy controls. Endothelial microvesicles demonstrated a positive correlation with endothelial and inflammatory markers, and clinical severity. Endothelial EVs may serve as potential biomarkers of endothelial activation and injury, although their levels may be influenced by the underlying condition and ECMO circuit.
Awad, 2014 [95] Endothelial dysfunction	Total: 85 neonates N: 45 ABO HDN N: 20 Rh HDN N: 20 Controls	ABO HDN: 37.8 (±1.1) weeks Rh HDN: 37.9 (±1.1) weeks; Control: 38 (±1.4) weeks	Pre- and post-exchange transfusion and/or phototherapy	Plasma (peripheral blood)	Endothelial cells Microparticles	EVs identity: CD144 Endothelial microparticles were measured at increased concentrations in ABO HDN vs. Rh HDN and healthy controls, in correlation with the severity of hyperbilirubinemia. The levels of endothelial microparticles declined substantially after intervention, especially after exchange transfusion. Endothelial microparticles could be used as markers of disease severity and suggest a possible long-term effect of ABO incompatibility on vascular status.
Zhu, 2019 [96] Endothelial dysfunction	Total: 72 neonates N: 29 ABO HDN N: 22 Rh HDN N: 21 Controls	ABO HDN: 38.1 (±1.2) weeks Rh HDN: 39 (±1.1) weeks; Controls: 40 (±1.5) weeks	Pre- and post-exchange transfusion and/or phototherapy	Plasma (peripheral blood)	Endothelial cells Microparticles	EV identity: CD144 Higher levels of endothelial microparticles were measured in ABO hemolytic disease vs. Rh hemolytic disease and healthy controls. Endothelial microparticles may be used as indicators of endothelial injury in ABO HDN.
Yücesoy, 2024 [97] Endothelial dysfunction	Total: 58 N: 33 neonates with non-hemolytic hyperbilirubinemia N: 25 Controls	Cases: 38.86 (±1.10) weeks Controls: 39.69 (±1.18) weeks	Cases: Pre- and post-phototherapy Controls: Single timepoint	Plasma (peripheral blood)	Endothelial cells, Platelets Microparticles	EVs identity: Annexin V for apoptotic MPs CD31 for Platelet-derived MPs CD144+ for Endothelial-derived MPs CD142 for TF expressing MPs Significantly higher levels of total apoptotic and endothelial-derived apoptotic microparticles in the study group vs. the control group, before and after phototherapy. Phototherapy exerted no impact on levels of total or any other type of microparticles.

Abbreviations: ECMO, Extracorporeal Membrane Oxygenation; EVs, extracellular vesicles; HDN, hemolytic disease of the newborn; MadCAM, mucosal vascular addressin cell adhesion molecule 1; MPs, microparticles; PECAM1, platelet and endothelial cell adhesion molecule 1; TF, tissue factor; VEGFR2, Vascular Endothelial Growth Factor Receptor 2.

**Table 6:** Extracellular vesicles (EVs) profiles and their potential as biomarkers in diverse neonatal and perinatal conditions.

Authors, Year Disease Entity	Study Population	Gestational Age (Weeks)	Sampling Timepoint	Biofluid	EVs Origin EVs Subtype and Size	Key Findings
Campello, 2015 [99] Preeclampsia	Total: 32 mother/infant pairs N: 16 neonates from preeclampsia pregnancies N: 16 neonates from non-preeclampsia pregnancies	Preeclampsia group: 38 (±6) weeks Non-preeclampsia group: 37 (±1) weeks	At birth	Plasma (UCB & peripheral blood)	Platelets, activated platelets, leukocytes, endothelial cells Microparticles	Total MPs (Annexin V), activated platelet-derived (CD62-P selectin), leukocyte-derived (CD45) and TF bearing MPs (CD142) were significantly increased in umbilical cord and peripheral blood in preeclamptic vs. normotensive women. The levels of platelet-derived MPs (CD61) were lower in preeclamptic women than controls, probably reflecting the reduced platelet count. The discrepancies were more intense in cord venous blood vs. peripheral venous blood, suggesting that microparticles are powerful procoagulants that counterbalance the immature fetal hemostatic mechanism.
Jia, [100] Preeclampsia	Total: 20 N: 10 neonates born after preeclampsia-complicated pregnancies N: 10 controls	Controls: 38.6 (±5.5) weeks Cases: 34.3 (±4.6) weeks	At birth	Plasma (UCB)	EV origin not determined Exosomes	Proteomic analysis: 29 differentially expressed proteins between the patient and control group that correlate with enzyme regulator activity, binding, extracellular region, cell part, biological regulation, cellular process and complement and coagulation cascades. Umbilical cord blood exosomes are probably involved in pathophysiology of preeclampsia through the complement and coagulation cascades.
Xueya, 2020 [101] Preeclampsia	Total: 38 mother/infant pairs N: 18 neonates from pregnancies complicated by preeclampsia N: 20 neonate controls	Preeclampsia group: 33.3 (±1.07) weeks Normotensive group: 39 (±0.22) weeks	At birth	Plasma (UCB)	EV origin not determined Exosomes	EVs identity: CD9 TSG-101, ALIX EV cargo of interest: miR-125a-5p Exosomal miR-125a-5p in preeclampsia downregulated angiogenesis by reducing VEGFA expression in placenta, directly related to the pathogenesis of preeclampsia
Kunte, 2023 [102] Neonatal adiposity	Total: 127 mother–infant pairs N: 51 lean infants N: 76 adipose infants	Mean GA lean group: 32.5 weeks Adipose neonates: 32 weeks	At birth	Plasma (UCB and maternal blood)	Adipocytes Small EVs	EVs identity: FABP4 EVs cargo: Several miRNAs were differentially expressed in ADsEVs in maternal and cord blood plasma samples at birth between lean and adipose neonates. The differentially expressed miRNAs were involved in adipogenesis-related pathways. The ADsEV miRNAs in mothers probably regulate fetal adiposity.
Alhamdan, 2025 [103] CHD surgery	Total: 10 infants N: 5 infants with organ dysfunction N: 5 infants with no organ dysfunction	Organ dysfunction group: 5.0 ± 5.1 months No organ dysfunction group: 5.9 ± 4.7 months	T0: after the induction of anesthesia T1: During the rewarming phase of CPB T2: Upon admission to the ICU following surgery T3: On postoperative day 1	Plasma (peripheral blood)	EV origin not recorded Exosomes	EVs identity: ALIX, TSG101 CD63, CD81, EpCAM Annexin A5, Flotillin-1, ICAM1, GM130 (cellular contamination marker) EV cargo: miR-200b-5p, miR-4800-5p, miR-363-3p, miR-483-5p were linked to organ dysfunction post-surgery. The EV miRNAs in the organ dysfunction group were mainly associated with upregulated apoptosis, proinflammatory pathways and NET formation across all timepoints, while complement and coagulation pathways were downregulated towards the end of surgery, indicating that EVs may serve as dynamic real-time biomarkers of the neonate’s biological response to significant surgical and inflammatory stress.

Abbreviations: ADsEVs, adipocyte-derived small EVs; CHD, Congenital Heart Disease; CPB, Cardiopulmonary Bypass; EpCAM, epithelial cell adhesion molecule; EVs, extracellular vesicles; GM130, Golgi matrix protein 130; ICAM1, intercellular adhesion molecule 1; ICU, Intensive Care Unit; MPs, microparticles; NET, neutrophil extracellular traps; TF: tissue factor; TSG-101; tumor susceptibility gene 101; UCB, umbilical cord blood; VEGFA, Vascular Endothelial Growth Factor A.

**Table 7:** The role of EVs as therapeutic tools.

Authors, Year	Disease Model	EVs Source	Isolation	EVs Dose	Route and Time of Administration	Outcome
	Bronchopulmonary Dysplasia					
Lee, 2012 [108]	Mice exposed to normobaric hypoxia (8.5% O_2_) for 48 h.	Mouse BM-derived MSCs and MSCs from hUC-Wharton’s Jelly (WJ-MSCs)	Ultrafiltration, PEG, size exclusion chromatography, UC	Multiple doses tested	Intravenous Multiple schemes and doses tested.	MSCs-derived exosomes’ administration exerted a protective role on the lung and ameliorated pulmonary hypertension through suppression of hyperproliferative pathways, such as STAT-3-mediated signaling induced by hypoxia.
Willis, 2018 [109]	Newborn term mice exposed to 75% O_2_ for 7 days	Human BM-derived and hUC-MSCs	Differential centrifugation, TFF, OptiPrep TM cushion density flotation	Cell equivalent: 0.5 × 10^6^ MSCs cultured over 36 h. NTA-corresponding dose: 7.2–8.5 × 10^8^ particles.	Intravenous once at PND4	MSC-exosomes improved alveolarization and lung architecture, as well as pulmonary hypertension. MSC-exosomes treatment modulated the lung transcriptome, including suppression of genes involved in inflammation and immune response. The proinflammatory “M1” state of macrophages was suppressed by MSC-exosomes, while an anti-inflammatory “M2-like” state was augmented both in vitro and in vivo.
Willis, 2021 [110]	Newborn term mice exposed to 75% O_2_ for 14 days	Human MSCs (umbilical cord, bone marrow)	Differential centrifugation, TFF, OptiPrep TM cushion density flotation	Cell equivalent: 0.5 × 10^6^ MSCs cultured over 36 h.	Intravenous once at PND4 MSC-EVs MSC-EVseducated bone marrow-derived myeloid cells	MSC-EVs improved alveolarization, blunted fibrosis and pulmonary vascular remodeling and inhibited apoptosis. A suppression of the inflammatory cell phenotype was also observed.
Wu, 2021 [111]	Newborn term rats exposed to 90% O_2_ from birth to PND 7.	Mouse BM-derived MSCs	Exosome isolation reagent & centrifugation for 1 h at 12,000× *g*	800 μg	Intravenous, time not recorded	MSC-EVs administration ameliorated hyperoxia-induced lung injury. The beneficial effect of MSC-EVs was partially attributed to the delivery of miR-425 into lung cells and upregulation of the PI3K/AKT axis.
Chaubey, 2018 [112]	Newborn term mice exposed to hyperoxia (>95% O_2_) for 4 days	Human MSCs (umbilical cord of preterm donors born at 25 weeks/30 weeks’ gestation)	10-fold concentrated CM & UC	Cell equivalent: 0.7 × 10^6^ MSCs cultured over 24 h. NTA-corresponding dose: 4.5 × 10^8^ particles derived from 25-week-old MSCs, and 2.88 × 10^7^ particles derived from 30-week-old MSCs.	Intraperitoneal at PND2 and 4	MSC-EVs administration ameliorated pulmonary infiltration and alveolar simplification. Improved pulmonary hypertension and a reversal of brain injury were also demonstrated. The therapeutic efficacy of MSC-EVs is probably mediated by TSG-6, an immunomodulatory glycoprotein.
Braun, 2018 [113]	Newborn term rats exposed to 85% O_2_ for 14 days	Rat BM-derived MSCs	UC	Protein amount: 15 μg/dose NTA-corresponding dose: 3.4 × 10^9^ particles.	Intraperitoneal daily PND1-15	MSC-EVs ameliorated hyperoxia-induced lung injury, induced pulmonary angiogenesis and reduced right ventricular hypertrophy. The beneficial effect was partially attributed to VEGF.
Ai, 2022 [114]	Newborn term rats exposed to 75% O_2_ for 14 days	hUC-derived MSCs	UC	Protein amount: 10 μg vs. 15 μg of protein/dose	Intraperitoneal once at PND4	A dose-dependent amelioration of alveolar simplification and fibrosis was demonstrated, associated with an improvement in vascular growth and pulmonary hypertension. MSC-EVs also suppressed the transdifferentiation of AT2 cells into AT1 cells induced by hyperoxia, probably through downregulation of WNT5a.
Zhou, 2022 [115]	BPD mouse model induced by in utero injection of LPS	hUC-MSCs	UC	20 μg/dose	Intratracheal on PND7	hUC-MSC-derived MVs improved lung function and enhanced alveolarization. An anti-inflammatory effect was also observed. The beneficial effects were associated with the PTEN/AKT and the MAPK Pathways.
Ahn, 2018 [116]	Newborn term rats exposed to 90% O_2_ for 14 days	hUC-MSCs	UC	20 μg/dose	Intratracheal once at PND5	MSC-derived EVs enhanced alveolarization and angiogenesis, while they suppressed apoptosis and proinflammatory cytokines. The beneficial effect was primarily mediated by VEGF transfer.
Porzionato, 2019, 2021 [117,118]	Newborn term rats exposed to 60% O_2_ for 14 days	hUC-MSCs	TFF	Cell equivalent: 2 × 10^6^ MSCs cultured over 36 h. NTA-corresponding dose: 8 × 10^8^, 4.5 × 10^8^, 3 × 10^8^, 1.5 × 10^8^ particles per gram of body weight at PND3, 7, 10, 21, respectively.	Intratracheal at PNDs 3, 7, 10, 21	MSC-EVs improved alveolarization and vascular remodeling, while they reduced fibrosis.
An, 2025 [119]	Newborn term rats exposed to 90% O_2_ for 14 days	Human Wharton’s jelly MSCs	TFF	1 × 10^9^ particles/dose	Intratracheal at PND2 and 5	MSC-EV administration improved alveolarization and lung angiogenesis, reduced lung inflammation and apoptotic cell death, mainly due to the miR-203a-3p cargo of EVs.
Sharma, 2022 [120]	Newborn term rats exposed to 85% O_2_ for 14 days	Wharton’s Jelly MSCs (WJ-MSCs) vs. BM-MSCs	UC	To compare BM- vs. WJ-MSC EVs, a single intra-tracheal injection of 12 × 10^8^ particles/g body weight was given. To determine the optimal dose of MSC EVs, 3 different doses were evaluated: 2 × 10^8^, 12 × 10^8^ and 60 × 10^8^ particles/g of body weight.	Intratracheal vs. intravenous once at PND3	Both BM- and WJ-MSC EVs demonstrated similar beneficial effects on lung structure and pulmonary hypertension, though hUC-MSC EVs were superior in preventing cardiac remodeling. The cardioprotective effects of hUC-MSC EVs persisted into adulthood.
Bellio, 2021 [121]	Hyperoxia-induced BPD rat model (85% O_2_ for 14 days)	Amniotic fluid (full-term cesarean sections)	Centrifugation, Filtration, UC	0.42 units Total dose—1 × 10^10^ EVs	Intratracheal at PND3	EV administration increased alveolar development and ameliorated pulmonary hypertension
Wang, 2023 [122]	Hypoxia-induced BPD rat model (40% O_2_ for 10 min + 1% O_2_ for 2 min, 12 times daily P1–P14)	Human BM-derived MSCs	UC	0.00012 units total dose—2 × 10^5^ EVs	Intraperitoneal per day, PND1-PND14	EV administration decreased alveolar simplification and oxidative stress. Pulmonary hypertension was ameliorated and respiratory efficiency was increased.
Lithopoulos, 2022 [123]	Term mice were injected intraperitoneally with LPS at PND7 or 8 and then ventilated (40% oxygen) for 8 h at PDN9 or 10.	hUC-MSCs	UC	Protein amount: 0.005 μg/g of body weight. NTA-corresponding dose: 1 × 10^6^ particles/g of body weight.	Intratracheal once at PND9 or 10 before ventilation	MESC-EVs increased anti-inflammatory cytokines and improved lung injury. Lung vessel density was augmented by MSC-EVs, while the self-renewal capacity of neuronal progenitor cells was also enhanced.
You, 2020 [124]	Newborn term rats exposed to 85% O_2_ for 14 days	hUC-MSCs	Serial centrifugation, UC	Protein amount: 20 μg/dose	Intratracheal once at PND7	MSC-EVs attenuated hyperoxia-induced lung injury, improved alveolarization, cell survival and angiogenesis, probably through the PTEN/Akt signaling pathway.
Zhu, 2022 [105]	Newborn term mice injected in utero with LPS and then exposed to 65% O_2_ from PND 3.5 until PDN7, PDN14 or PDN28	Preterm and term hAECs	UC	Protein amount: 10 μg/dose	Intravenous at PDN4	Term hAEC-EVs improved tissue-to-air space ratio and secondary septal crest density, decreased lung inflammation, reduced peripheral pulmonary arterial remodeling and airway hyper-responsiveness, while they prevented pulmonary hypertension and right ventricular hypertrophy. Preterm hAEC-EVs demonstrated diminished therapeutic effect.
Xu, 2025 [125]	Newborn term mice exposed to 85% O_2_	hUC-MSCs	UC	NTA-corresponding dose: 2 × 10^6^ particles/g of body weight	Intranasal, every other day from postnatal day PND1 to PND14, total seven doses.	MSC-EVs administration promoted alveolarization, restored efficient lung ventilation, alleviated inflammation and repaired pulmonary vascular injury.
Abele, 2022 [126]	Rat model of chorioamnionitis-induced BPD (rats prenatally exposed to endotoxin at embryonic day 20)	Human BM MSCs	Differential centrifugation, TFF, OptiPrep TM cushion density flotation	Cell equivalent: 0.25 × 10^6^ MSCs cultured over 36 h. NTA-corresponding dose: 4.25 × 10^8^ particles.	Intra-amniotic at embryonic day 20, along with endotoxin injection	Antenatal administration of MSC-EVs reduced inflammatory cytokines, preserved distal lung growth and mechanics.
Zhou, 2022 [127]	Newborn term rats exposed to 85% O_2_ from PND1 to PND7	Human breast milk-derived EVs	UC	0.133 units	Intragastric administration at PND7	Human breast milk-derived EVs downregulated cell apoptosis and repaired hyperoxia-induced AT II cell damage.
Willis, 2020 [128]	Newborn term mice exposed to 75% O_2_ for 14 days	Human WJ-derived MSCs	Differential centrifugation, TFF, OptiPrep TM cushion density flotation	Cell equivalent: 0.5 × 10^6^ MSCs (early intervention), 1 × 10^6^ MSCs (late intervention) cultured over 36 h. NTA-corresponding dose: 6 × 10^8^ and 1.7 × 10^9^ particles, respectively.	Intravenous Single dose at PND4 (“early intervention”) Single dose at PND18 or multiple doses weekly at PND18, 25, 32, 39 (“late intervention”)	MSC-EV administration (both early and late interventions) improved alveolar simplification and vascular remodeling. Decreased pulmonary fibrosis was also demonstrated, along with improvement in exercise capacity testing and pulmonary hypertension.
Zhong, 2023 [129]	Newborn term mice exposed to 75% O_2_ from PND1 until PND14	Human umbilical cord blood MSCs	UC	0.000063 units Total dose: 15 × 10^5^ EVs	Intraperitoneal for 3 days starting from PND4	MSC-EV alleviated hyperoxia-induced lung injury and promoted vascular growth. The beneficial effect was partially attributed to miR-185-5p expression.
Wu, 2022 [130]	Newborn term mice exposed to 95% O_2_ for 3 days	Mouse adipose-derived MSCs	UC	Protein amount: 30 ng vs. 300 ng/dose	Intratracheal once at PND1	Reduced lung inflammation, oxidative stress and apoptosis. The therapeutic effect was partially mediated through miR-21-5p expression.
	Perinatal Brain Injury					
Sisa, 2019 [131]	The Rice–Vannucci model *	Human BM MSCs	UC	1.1 units Total dose—1.25 × 10^9^ EVs	Intranasal	MSC-EV administration decreased microglial activation and cell death, as well as volume loss. An improvement in behavioral outcomes was also observed.
Labusek, 2023 [132]	The Rice–Vannucci model	Immortalized human BM MSCs	PEG, UC	0.03 units Total dose—2.7 × 10^8^ EVs	Intranasal on days 1, 3, 5 post-HI	MSC-EV administration reduced astrogliosis and microgliosis, endothelial activation and leukocyte infiltration. The pro-inflammatory cytokines were downregulated, while the anti-inflammatory cytokines were upregulated. Oligodendrocyte maturation was enhanced and a protective effect on HI-induced brain atrophy was demonstrated.
Labusek, 2023 [133]	The Rice–Vannucci model	Immortalized human BM MSCs	PEG, UC	0.03 units Total dose—2.7 × 10^8^ EVs	Intranasal on days 1, 3, 5 post-HI	MSCs-EV administration combined with hypothermia resulted in improved long-term neurodevelopmental outcome, protected from neuronal, axonal and vascular injury and prevented endothelial activation and leukocyte infiltration. Also, the combined therapy reduced microglial activation, astrogliosis and pro-inflammatory cytokine expression, while upregulation of anti-inflammatory cytokines and trophic growth factors was demonstrated.
Kaminski, 2020 [134]	The Rice–Vannucci model	Human BM MSCs	Sequential centrifugation, PEG and UC	0.03 units Total dose—2.7 × 10^8^	Intraperitoneal at days 1, 3, 5 post-HI	MSC-EV administration protected from striatal tissue loss, decreased micro- and astroglial activation, downregulated pro-inflammatory cytokines and upregulated anti-inflammatory cytokines and neural growth factors. Oligodendrocyte maturation and myelination were also improved.
Ophelders, 2016 [135]	Preterm ovine model of hypoxic–ischemic injury (HI induced by umbilical cord occlusion)	Human BM MSCs	MSC-CM filtration, PEG, low-speed centrifugation	Total dose: 4.0 × 10^7^ cell equivalents divided into two boluses of 2.0 × 10^7^ cell equivalents.	In utero intravenous. The first bolus was administered 1 h and the second bolus was administered 4 days after the HI insult.	MSC-EV administration reduced the total number and duration of seizures, preserved baroreceptor reflex sensitivity, while a tendency to prevent hypomyelination was demonstrated.
Turovsky, 2022 [136]	The Rice–Vannucci model	Human placenta	UC	Dose: 20 μL MSC-EV suspension containing 1.6 ± 0.2 × 10^11^ particles/mL of MSC-EV.	Intranasal, daily for two weeks, excluding weekends, for a total of 10 treatments.	MSC-EV administration mitigated HI-induced brain injury and promoted the recovery of sensorimotor functions. The beneficial effect was mediated by the PI3K/AKT pathway.
Gussenhoven, 2019 [137]	Preterm fetal ovine model with HI induced by transient umbilical cord occlusion	Human BM MSCs	PEG, low-speed centrifugation and UC	Two boluses of MSC-EVs from 2 × 10^7^ cell equivalents.	In utero intravenous at 1 h and 4 days following HI insult.	MSC-EV administration protected the blood–brain barrier via FPR signaling. MSC-EVs contain the potent FPR agonist Annexin A1.
Hu, 2021 [138]	BV-2 cells were subjected to oxygen-glucose deprivation/reoxygenation (OGD/R) to induce microglial ischemia/reperfusion (I/R) in vitro	hUC-MSCs	UC	Not applied	Not applied	MSC-EV ameliorated neuronal injury by promoting FOXO3a expression to enhance mitophagy, thus protecting microglia from I/R-induced pyroptosis.
Du, 2021 [139]	The Rice–Vannucci model	Rat primary astrocytes	UC	0.0024 units Total dose—2.5 µg of EV protein	Intraperitoneal	Astrocyte-derived EV administration improved neurobehaviors and reduced cerebral infarction, neuronal apoptosis, oxidative stress and neuroinflammation. The beneficial effect could be attributed to miR-17-5p in astrocyte-derived EVs that protects neonatal rats from hypoxia-induced brain injury via inhibiting BNIP-2 expression.
Xin, 2020 [140]	The Rice–Vannucci model	Not determined	UC	100 μg	Intracardial at 24 h following HI exposure	MSC-EV administration ameliorated acute brain injury and neuroinflammation. MSC-EVs can be taken up by neurons and microglia and exert a neuroprotective effect through the delivery of miR-21a-5p.
Joerger-Messerli, 2018 [141]	An in vitro model of oxygen–glucose deprivation/reoxygenation (OGD/R)	hWJ-MSCs	Serial centrifugation	Not applied	Not applied	The administration of hWJ-MSC-EVs can potentially prevent and resolve HI-induced apoptosis in neuronal cells, probably through the let-7-5p miR family contained in hWJ-MSC-EVs.
Luo, 2022 [142]	Rat models of middle cerebral artery occlusion	Neural stem cells	UC	Not determined	Intravenous	Neural stem cell EVs significantly reduced the infarction area and suppressed apoptosis through transfer of miR-150-3p, which targets caspase-2.
Luo, 2022 [143]	The Rice–Vannucci model	mice BM-MSCs	Serial centrifugation, filtration, UC	1.2 units total dose—2 × 10^9^ EVs	Intranasal	MSC-EV administration decreased neuronal death and neuroinflammation. The beneficial effect was mediated by miR-93 suppressing the JMJD3/p53/KLF2 axis
Chu, 2020 [144]	The Rice–Vannucci model	Rat BM-MSCs (H2S preconditioning)	Centrifugation, filtration, ultrafiltration and EVs isolation kit (qEV, iZonScience)	0.06 units total dose—1.5 × 10^8^ EVs	Intracardial	MSC-EV administration decreased brain tissue loss and levels of pro-inflammatory mediators, while anti-inflammation was augmented. An associated improvement in long-term cognitive and memory functions was observed. The beneficial effect was mediated by miR-7b-5p expression.
Thomi, 2019 [145]	A model of combined hypoxia–ischemia and inflammation	Human WJ-MSCs	Serial centrifugation and UC	50 mg/kg	Intranasally	MSC-EV administration rescued normal myelination and mature oligodendroglial and neuronal cell counts, while a significantly improved learning ability was demonstrated.
Drommelschmidt, 2017 [146]	Inflammation-induced brain injury	Human BM-MSCs	PEG, UC	2 doses of 1 × 10^8^ cell equivalents/kg	Intraperitoneal	MSC-EV administration significantly reduced inflammation-induced neuronal cellular degeneration and microgliosis. Additionally, reactive astrogliosis was prevented, and myelination deficits and microstructural abnormalities of the white matter were restored. An improvement in long-lasting cognitive functions was demonstrated.
Thomi, 2019 [147]	A model of combined hypoxia–ischemia and inflammation	Human WJ MSCs	Serial centrifugation and UC	50 mg/kg	Intranasal	MSC-EV administration reduced neuroinflammation and microgliosis.
Ahn, 2022 [148]	IVH rodent model	hUC-MSCs	UC	20 μg of EVs	Intraventricular at PND 6	MSC-EV administration attenuated neuronal cell death, astrogliosis and inflammatory response, enhanced neurogenesis, halted the progression to post-hemorrhagic hydrocephalus and improved the behavioral test performance. The beneficial effect was primarily mediated by BDNF carried by EVs.
Pathipati, 2021 [149]	Perinatal rodent stroke model	Mice BM-MSCs	Sequential centrifugation, filtration, ExoQuick TC Ultra	1 Dose of 1 μg/μL or 5 μg/μL	Intracerebroventricular or intranasal	MSC-EV administration significantly reduced injury volume, in part via modulatory effects on microglial cells. A reduction in caspase-3-dependent apoptotic cell death was also identified.
	Necrotizing Enterocolitis					
Rager, 2016 [150]	NEC-induced preterm mouse model	Mice BM-MSCs	In vivo: P100 PureExo Exosome Isolation kit; in vitro: serial centrifugation & UC	2.5 × 10^9^ EVs	Intraperitoneal	MSC-EV administration reduced the incidence and severity of NEC and preserved gut barrier function.
McCulloh, 2018 [151]	NEC rodent model	Rat AF-MSC, BM-MSC, AF neural SCs, enteric neural SCs	Differential UC	Varying doses	Intraperitoneal	MSC-EV administration reduced NEC incidence and intestinal injury. The best results were identified at doses of 8 × 10^7^ or 4.0 × 10^8^ EVs/50 μL.
Li, 2020 [152]	NEC-induced preterm mouse model	Rat amniotic fluid stem cells	ExoQuick	2 Doses of AFSC-EVs derived from 200 μL of CM of 2 × 10^6^ AFSCs	Intraperitoneal at PND6 and PND7 or at PND3 and PND4 prior to NEC induction	EV administration ameliorated NEC intestinal injury by activating the Wnt signaling pathway. Intestinal recovery from NEC was also promoted by increasing cellular proliferation and reducing inflammation. A regenerative effect towards a normal intestinal epithelium was demonstrated. A preventive effect, however, was not observed.
Hu, 2023 [153]	NEC mouse model	Human AFSC, HBM	Serial centrifugation, UC	5 μg	A gavage of EVs at PND 8–10, three times a day.	EVs derived from AFSC and HBM attenuated intestinal injury, reduced systemic and local inflammation, as well as NEC-related brain injury. AFSC-EVs exerted their action through inhibition of the inflammatory response of intestinal epithelial cells, while HBM-EVs regulated the migration of intestinal epithelial cells.
	Retinopathy of prematurity					
Xu, 2019 [154]	Mice model of oxygen-induced retinopathy	Microglial cells	UC	1 mL of microglial exosome solution (1 mg/mL) in each eye	Intravitreal	EV administration resulted in smaller avascular areas and fewer neovascular tufts, as well as reduced photoreceptor apoptosis. The expression of VEGF and TGF-β was downregulated. The beneficial effect was mainly attributed to miR-24-3p in EVs.
Moisseiev 2017 [155]	Mice model of oxygen-induced retinopathy	Human BM-MSCs	Serial centrifugation, TFF, VivaSpin filtration column	20 µg in 1 µL	Intravitreal at PND12	EV administration partially preserved retinal vascular flow, improved retinal thinning and reduced retinal neovascularization.
Mathew, 2019 [156]	Rat model of retinal ischemia	Human BM-MSCs	Sequential centrifugation, ultrafiltration & Exo Quick-TC EV	4 μL of 1 × 10^9^ particles/mL	Intravitreally 24 h post-injury in both the ischemic and non-ischemic eyes	MSC-derived EVs administration reduced cell death and loss of cell proliferation, suppressed neuroinflammation and apoptosis, while functional recovery was augmented.

Abbreviations: AF, amniotic fluid; AFSC, Amniotic Fluid Stem Cells; AT II, Alveolar Type II cells, BDNF, brain-derived neurotrophic factor; BM, bone marrow; BPD, bronchopulmonary dysplasia; CM, conditioned medium; EVs, extracellular vesicles; hAECs, human amniotic epithelial cells; HI, hypoxic insult; HBM, human breast milk; hUC, human umbilical cord; IVH, intraventricular hemorrhage; LPS, lipopolysaccharide; MSCs, mesenchymal stem cells; MVs, microvesicles; NEC, necrotizing enterocolitis; PEG, polyethylene glycol; PND, postnatal day; SCs, stem cells; STAT3, signal transducer and activator of transcription 3; TGF-b, transforming growth factor b; TFF, tangential flow filtration; TSG-6, tumor necrosis factor alpha stimulated gene-6; UC, ultracentrifugation; VEGF, vascular endothelial growth factor; WJ, Wharton’s Jelly. The Rice–Vannucci model *: An experimental model of hypoxic–ischemic encephalopathy, including the following steps: common carotid artery ligation, brief post-operative normothermia, exposure to hypoxia, post-hypoxic normothermia [157].

**Table 8:** Summary of clinical trials of EVs in neonatal diseases.

Study	Location	Disease Entity	Study Characteristics	Gestational Age	EVs Source	Route of Administration	Dosage	Frequency	Goal	Time Frame	Current Status
Administration of Extracellular Vesicles From Donor Human Milk in Preterm Infants (AdVEMPrem study) (NCT07402083)	Spain	NEC prevention	Single-center, safety and tolerability study	<32 weeks	Donor human milk	Oral	Not determined yet	Not determined yet	Feeding tolerance, growth and neurodevelopmental outcome will be assessed, along with metabolism and stress markers. Safety profile: recording of any adverse events, like NEC, metabolic or renal impairment, cholestasis, sepsis.	From enrollment and extends up to 24 months of corrected age.	Recruiting
Safety and Efficacy of MSC-EVs in the Prevention of BPD in Extremely Preterm Infants (EVENEW) (NCT06279741)	Belgium, Italy	BPD	Multicenter, double-blind, randomized, placebo-controlled, dose-finding safety and efficacy study	23–27^+6^ weeks	Umbilical cord mesenchymal stromal cells	Intratracheal	Phase 1: Three dose levels (low dose, medium dose and high dose). Phase 2: Based on phase 1 study results	Phase 1: One or three administrations Phase 2: Two doses	Evaluation of incidence and severity of BPD. Recording of adverse events, assessment of lung ultrasound score, respiratory morbidity and neurodevelopmental outcome.	Until 2 years of corrected age	Recruiting
The Pilot Experimental Study of the Neuroprotective Effects of Exosomes in Extremely Low Birth Weight Infants (NCT05490173)	Moscow, Russia	Prematurity-related brain injury	Single-center, single blind, randomized, controlled, safety and efficacy study	25–27^+6^ weeks	Mesenchymal stromal cells	Intranasal	1 dose will be obtained from a daily conditioned culture medium of 120 million MSCs suspended in 500 µL of phosphate buffer in each nostril at 50 µL with an interval of 2–3 min	The therapeutic course will consist of 5 instillations with an interval of 1 day.	Primary outcome: Neurodevelopmental outcome. Incidence of IVH, PVL, brain injury. Secondary outcome: survival with and without major neonatal morbidity, including severe ROP, BPD, NEC. Safety profile: allergic reactions, damage to nasal mucosa or other adverse events.	Until 36 months of age	Not yet enrolling
Safety and Efficacy Study of Neural Progenitor Cell Transplantation and Paracrine Factors From Human Mesenchymal Stem Cells to Treat Newborn With Hypoxic–ischemic Encephalopathy (NCT02854579)	Beijing, China	HIE	Single-center, randomized, controlled, safety and efficacy study	≥34 weeks	Human mesenchymal stem cells	Intrathecally	0.5 mL of concentrated paracrine factors of human mesenchymal stem cells	At 12 h, 24 h, 48 h after birth	Neurodevelopmental outcome. Record of any adverse events.	Until 18 months	Recruiting

Abbreviations: BPD, bronchopulmonary dysplasia; EVs, extracellular vesicles; HIE, hypoxic–ischemic encephalopathy; IVH, intraventricular hemorrhage; MSCs, mesenchymal stromal cells; NEC, necrotizing enterocolitis; PVL, periventricular leukomalacia; ROP, retinopathy of prematurity.

## Data Availability

No new data were created or analyzed in this study. Data sharing is not applicable to this study.
